# Engineering *Saccharomyces cerevisiae* for enhanced (–)-α-bisabolol production

**DOI:** 10.1016/j.synbio.2023.01.004

**Published:** 2023-01-20

**Authors:** Yinkun Jiang, Lu Xia, Song Gao, Ning Li, Shiqin Yu, Jingwen Zhou

**Affiliations:** a Engineering Research Center of Ministry of Education on Food Synthetic Biotechnology, Jiangnan University, 1800 Lihu Road, Wuxi, Jiangsu, 214122, China; b Science Center for Future Foods, Jiangnan University, 1800 Lihu Road, Wuxi, Jiangsu, 214122, China; c Key Laboratory of Industrial Biotechnology, Ministry of Education and School of Biotechnology, Jiangnan University, 1800 Lihu Road, Wuxi, Jiangsu, 214122, China; d Jiangsu Province Engineering Research Center of Food Synthetic Biotechnology, Jiangnan University, Wuxi, 214122, China

**Keywords:** Metabolic engineering, (–)-α-bisabolol, Fusion expression, Transporter, *Saccharomyces cerevisiae*

## Abstract

(–)-α-Bisabolol is naturally occurring in many plants and has great potential in health products and pharmaceuticals. However, the current extraction method from natural plants is unsustainable and cannot fulfil the increasing requirement. This study aimed to develop a sustainable strategy to enhance the biosynthesis of (–)-α-bisabolol by metabolic engineering. By introducing the heterologous gene *MrBBS* and weakening the competitive pathway gene *ERG9*, a *de novo* (–)-α-bisabolol biosynthesis strain was constructed that could produce 221.96 mg/L (–)-α-bisabolol. Two key genes for (–)-α-bisabolol biosynthesis, *ERG20* and *MrBBS*, were fused by a flexible linker (GGGS)_3_ under the *GAL7* promoter control, and the titer was increased by 2.9-fold. Optimization of the mevalonic acid pathway and multi-copy integration further increased (–)-α-bisabolol production. To promote product efflux, overexpression of *PDR15* led to an increase in extracellular production. Combined with the optimal strategy, (–)-α-bisabolol production in a 5 L bioreactor reached 7.02 g/L, which is the highest titer reported in yeast to date. This work provides a reference for the efficient production of (–)-α-bisabolol in yeast.

## 1 Introduction

**Fig. 1:**
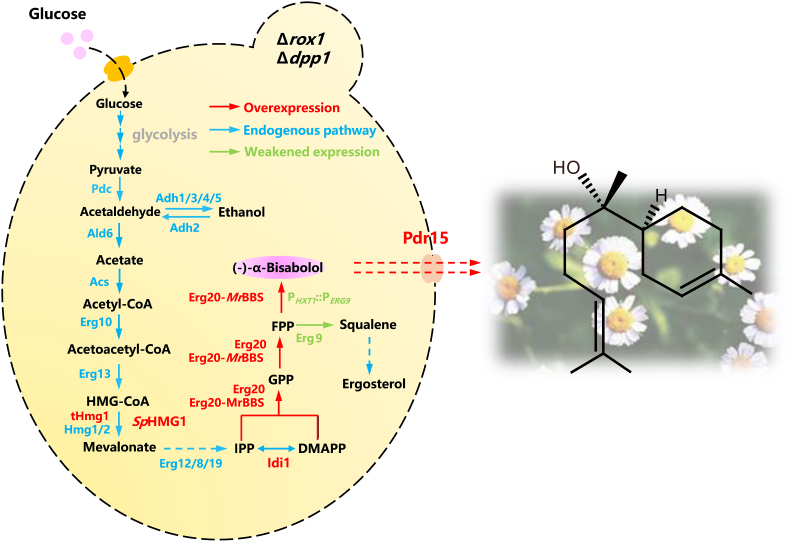
Metabolic engineering of *S. cerevisiae* to synthesize (–)-α-bisabolol. The biosynthesis pathway of (–)-α-bisabolol in *S. cerevisiae*. Abbreviations for enzyme and intermediate metabolite names are listed as follows: Pdc, pyruvate decarboxylase; Dpp1, diacylglycerol pyrophosphate phosphatase; Rox1, heme-dependent repressor of the hypoxic gene; Adh, alcohol dehydrogenase; Acs1, acetyl-CoA synthetase; Erg10, acetyl-CoA thiolase; Erg13, HMG-CoA synthase; Hmg1/2, HMG-CoA reductase; Erg12, mevalonate kinase; Erg8, mevalonate pyrophosphate kinase; Erg19, mevalonate pyrophosphate decarboxylase; Idi1, isopentenyl pyrophosphate isomerase; Erg20, farnesyl pyrophosphate synthase; *Mr*BBS, (–)-α-bisabolol synthase; Erg9, squalene synthase; Pdr15, plasma membrane ATP-binding cassette (ABC) transporter; IPP, isopentenyl pyrophosphate; DMAPP, dimethylallyl pyrophosphate; GPP, geranyl pyrophosphate; FPP, farnesyl pyrophosphate.

Sesquiterpenoids are a class of natural organic compounds produced by condensing two isopentenyl pyrophosphate (IPP) units and a dimethylallyl pyrophosphate (DMAPP) unit [1]. Because of their strong aroma and anti-inflammatory effects, they have been widely used [2]. (–)-α-Bisabolol (C_15_H_26_O) is a monocyclic sesquiterpene alcohol that was isolated and extracted from chamomile [3]. Due to its anti-inflammatory, bactericidal, antibacterial, skin soothing and moisturizing properties [4], it has been used as an ingredient in pharmaceuticals and cosmetics. In the future, (–)-α-bisabolol may be applied to clinical practice because of its analgesic function [5]. Moreover, (–)-α-bisabolol has low physiology toxicity [6], and it has great potential to become a widely used health product.

(–)-α-Bisabolol exists naturally in *Eremanthus erythropappus* and *Matricaria recutita* [7]. The leaves of *E. erythropappus* have been used to produce (–)-α-bisabolol by extraction and distillation [8]. (–)-α-Bisabolol was also prepared by chemical synthesis, which required additional purification of (–)-α-bisabolol from its diastereomers, (+)-α-bisabolol and (±)-epi-α-bisabolol, formed during the synthetic process [9]. The low productivity of plant extraction and the low specificity of chemical catalysis limit the range of applications of (–)-α-bisabolol. In recent years, the advantages of using microbial cell factories to produce (–)-α-bisabolol have become increasingly attractive [10]. While industrial production remained many challenges due to the low titer of (–)-α-bisabolol. As for the protocol for the biosynthesis of (–)-α-bisabolol in *Saccharomyces cerevisiae* using glucose as a cheap carbon source to produce acetyl-CoA, which enters the mevalonic acid pathway (MVA pathway) to produce farnesyl pyrophosphate (FPP). Then, FPP is converted to (–)-α-bisabolol by a (–)-α-bisabolol synthase from *M. recutita* (*Mr*BBS) (Fig. 1) [11].

Recently, considerable efforts have focused on increasing the production of (–)-α-bisabolol in microbial cell factories, but there still exist many limitations and challenges. Overexpression of the heterologous mevalonate pathway genes (*mvaE*, *mvaS*, *mvaK1*, *mvaK2*, *mvaD* and *idi*) and farnesyl pyrophosphate synthetase gene *ispA* in *Escherichia coli* could improve the (–)-α-bisabolol titer to 9.1 g/L [12]. The efficient (–)-α-bisabolol synthase *Cc*BOS from *C. cardunculus* var. *Scolymus* was tested, which increased the titer to 23.4 g/L in *E. coli* [13]. In contrast to *E. coli*, fungi are less susceptible to phage infection. Optimization of the MVA pathway is the key to (–)-α-bisabolol production in yeast. Shi et al. overexpressed the entire MVA pathway genes in *Y. lipolytica* to increase the metabolic flux to FPP and optimize the copy number of *MrBBS* and *tHMG1*, resulting in the production of 4.4 g/L in a 5 L bioreactor. This was the highest production in yeast reported to date [14]. A recent study showed that overexpression of *tHMG1*, *ERG10*, and *ACS1* lead to a 13-fold increase in (–)-α-bisabolol biosynthesis in *S. cerevisiae*, but the final production was only 124 mg/L [11]. Compared with *Y. lipolytica*, the highest titer of (–)-α-bisabolol in *S. cerevisiae* was markedly lower. Therefore, the bottleneck in the synthesis of (–)-α-bisabolol by *S. cerevisiae* needs to be solved.

To search for bottleneck steps of (–)-α-bisabolol biosynthesis in *S. cerevisiae*, a series of (–)-α-bisabolol product strains were constructed and verified. First, the heterologous gene *MrBBS* was introduced and the competitive pathway gene *ERG9* was weakened. Then, a flexible linker was used to fuse the key genes *ERG20* and *MrBBS*. Next, the effects of MVA pathway modulation and multi-copy integration of the key limiting genes were studied. Additionally, the effect of different transporters on the transport of (–)-α-bisabolol was tested. Finally, the production of (–)-α-bisabolol in a 5 L bioreactor reached 7.02 g/L. This is the highest titer of (–)-α-bisabolol in yeast reported to date. This sustainable method has the potential for industrial (–)-α-bisabolol production.

## 2 Materials and methods

### 2.1 Strains, plasmids and gene

The SQ14 strain was constructed in previous work [15]. The strains constructed in this study are shown in Table 2. *E. coli* JM109 was used for propagation and plasmid construction. The plasmid pY26-TEF-GPD with *URA3* selection markers was used for gene expression in *S. cerevisiae*. pMD19T-Simple (TaKaRa, Dalian, Liaoning, China) was used for gene cloning. The plasmids constructed in this study are shown in Table S1. The primers used to construct the plasmids were synthesized by Sangon Biotech (Shanghai, China). The linker sequences are listed in Table S2. The *MrBBS* gene was codon-optimized and synthesized by Azenta (Suzhou, Jiangsu, China), and the sequence is shown in Table S2.

**Table 1:** (–)-α-Bisabolol production in yeasts.

Chassis strain	Strategy	Titer (mg/L)	Productivity (mg L^−1^h^−1^)	Fermentation method	Reference
*Y. lipolytica*	*SQS1* was weakened, *MrBOS*, *tHMG1*, *ERG20* and *POT1* were overexpressed	364.23	3.04	Shake flask	[10]
*Y. lipolytica*	Iteratively integrating an entire MVA pathway, optimizing the copy number of *tHMG1* and *MrBBS*	4400	26.20	5L-Bioreactor	[14]
*S. cerevisiae*	(–)-α-Bisabolol synthase (*Mr*BBS) was overexpressed	8	–	Shake flask	[7]
*S. cerevisiae*	*tHMG1*, *ERG10* and *ACS1* were overexpressed	124	2.10	Shake flask	[11]
*S. cerevisiae*	–	7020	58.50	5L-Bioreactor	This study

**Table 2:** Strains used in this part of the experiment.

Strains	Host strain	Description	Source
CEN. PK2-1D		*MATα, his3Δ1, leu2-3_112, ura3-52, trp1-289, MAL2-8*^*c*^*, SUC2*	[43]
SQ14	CEN. PK2-1D	*MATα*, *his3Δ1*, *ura3-52*, *trp1-289*, *MAL2-8*^c^, *SUC2*, Ty2::P_*TEF1*_-*tHMGR*-P_*GAL7*_-*IDI1*-*KlLEU2*, Δ*gal2*::*NADH-HMG1*, ΔP_*INO2*_:: P_*PGK1*_, Δ*tat1*::*ACL*	[15]
BS2	SQ14	Δ*spr1*::P_*GAL10*_-*MrBBS*-T_*TAT1*_	This study
BS3	BS2	ΔP_*ERG9*_::P_*HXT1*_	This study
BS3-MrBBS	BS3	pY26-P_*GAL10*_-*MrBBS*-T_*TAT1*_	This study
BS3-BB3	BS3	pY26-P_*GAL10*_-*ERG20*-P_*GAL10*_-*MrBBS*-T_*TAT1*_	This study
BS3-BB4	BS3	pY26-P_*GAL10*_-*ERG20-*GGGS*-MrBBS*-T_*TAT1*_	This study
BS3-BB5	BS3	pY26-P_*GAL10*_-*MrBBS-*GGGS*-ERG20*-T_*TAT1*_	This study
BS3-BB6	BS3	pY26-P_*GAL10*_-*ERG20-*(GGGS)_2_*-MrBBS*-T_*TAT1*_	This study
BS3-BB7	BS3	pY26-P_*GAL10*_-*ERG20-*(GGGS)_3_*-MrBBS*-T_*TAT1*_	This study
BS3-BB8	BS3	pY26-P_*GAL10*_-*MrBBS*-(GGGS)_2_*-ERG20*-T_*TAT1*_	This study
BS3-BB9	BS3	pY26-P_*GAL10*_-*MrBBS*-(GGGS)_3_-*ERG20*-T_*TAT1*_	This study
BS3-BB10	BS3	pY26-P_*INO1*_-*ERG20*-(GGGS)_3_-*MrBBS-*T_*TAT1*_	This study
BS3-BB11	BS3	pY26-P_*TDH3*_-*ERG20*-(GGGS)_3_-*MrBBS*-T_*TAT1*_	This study
BS3-BB12	BS3	pY26-P_*GAL7*_-*ERG20*-(GGGS)_3_-*MrBBS*-T_*TAT1*_	This study
BS3-BB13	BS3	pY26-P_*GAL2*_*-ERG20*-(GGGS)_3_-*MrBBS*-T_*TAT1*_	This study
BS3-BB14	BS3	pY26-P_*ERG1*_-*ERG20*-(GGGS)_3_-*MrBBS*-T_*TAT1*_	This study
BS3-BB15	BS3	pY26-P_*RPS5*_-*ERG20*-(GGGS)_3_-*MrBBS*-T_*TAT1*_	This study
BS3-1	BS3	Δ*dpp1*	This study
BS3-2	BS3	Δ*dpp1*::P_*GAL10*_-*ERG10*- T_*TAT1*_	This study
BS3-3	BS3	Δ*dpp1*::P_*GAL10*_-*ERG20*-T_*TAT1*_	This study
BS3-4	BS3	Δ*dpp1*::P_*GAL2*_-*UPC2*^*G888A*^-T_*ADH1*_-P_*GAL10*_-*ERG20*-T_*TAT1*_	This study
BS3-5	BS3-3	Δ*rox1*	This study
BS3-6	BS3-3	Δ*ypl062w*	This study
BS3-7	BS3-3	ΔP_*ADH2*_::P_*TEF1*_	This study
BS3-1-BB13	BS3-1	pY26-P_*GAL2*_*-ERG20*-(GGGS)_3_-*MrBBS*-T_*TAT1*_	This study
BS3-2-BB13	BS3-2	pY26-P_*GAL2*_*-ERG20*-(GGGS)_3_-*MrBBS*-T_*TAT1*_	This study
BS3-3-BB13	BS3-3	pY26-P_*GAL2*_*-ERG20*-(GGGS)_3_-*MrBBS*-T_*TAT1*_	This study
BS3-4-BB13	BS3-4	pY26-P_*GAL2*_*-ERG20*-(GGGS)_3_-*MrBBS*-T_*TAT1*_	This study
BS3-5-BB13	BS3-5	pY26-P_*GAL2*_*-ERG20*-(GGGS)_3_-*MrBBS*-T_*TAT1*_	This study
BS3-6-BB13	BS3-6	pY26-P_*GAL2*_*-ERG20*-(GGGS)_3_-*MrBBS*-T_*TAT1*_	This study
BS3-7-BB13	BS3-7	pY26-P_*GAL2*_*-ERG20*-(GGGS)_3_-*MrBBS*-T_*TAT1*_	This study
BS5	BS3-5	Ty3::P_*GAL7*_-*ERG20*-(GGGS)_3_*-MrBBS*-T_*TAT1*_-*KlURA3deg*	This study
BS5-1	BS5	Δ*pdr12*::*HIS3*	This study
BS5-2	BS5	Δ*pdr1*::*HIS3*	This study
BS5-3	BS5	Δ*pdr5*::*HIS3*	This study
BS5-4	BS5	Δ*snq2*::*HIS3*	This study
BS5-5	BS5	Δ*aus1*::*HIS3*	This study
BS5-6	BS5	Δ*pdr15*::*HIS3*	This study
BS5-7	BS5	Δ*pdr11*::*HIS3*	This study
BS5-8	BS5	Δ*ste6*::*HIS3*	This study
BS5-9	BS5	Δ*yor1*::*HIS3*	This study
BS5-10	BS5	Δ*pdr10*::*HIS3*	This study
BS5-11	BS5	Δ*war1*::*HIS3*	This study
BS5-12	BS5	Δ*msn4*::*HIS3*	This study
BS5-13	BS5	Δ*mot3*::*HIS3*	This study
BS5-14	BS5	Δ*pdr3*::*HIS3*	This study
BS5-15	BS5	Δ*aro80*::*HIS3*	This study
BS5-16	BS5	Δ*yrr1*::*HIS3*	This study
BS6	BS5	Δ*pdr15*::P_*TEF1*_-*PDR15*-T_*ADH2*_	This study
BS6-2	BS5	Δ*pdr3*::P_*TEF1*_-*PDR3*-T_*ADH2*_	This study

### 2.2 Strains construction

A Vazyme (Nanjing, China) high-fidelity Phusion DNA polymerase was used for amplifying DNA fragments. DNA fragments were purified and Gibson assembly was used to complete plasmid construction [16]. A Sangon Biotech (Shanghai, China) Seamless Cloning Kit was used for Gibson assembly. After cloning, all extracted plasmids were verified by Sangon Biotech (Shanghai, China). This study used the CRISPR-Cas9 system for single-gene editing [17], and the sgRNAs were designed using the website (https://www.benchling.com/). Both the promoter and terminator used in this study used the endogenous genetic elements of *S. cerevisiae*. A Frozen-EZ Yeast Transformation II Kit (Zymo Research, Los Angeles, CA, USA) was used for plasmid transformation and genome integration in *S. cerevisiae*. After transformation, cells were plated on Yeast Nitrogen Base (YNB)-defective plates (Sangon Biotech, Shanghai, China). and cultivated for 3–5 days at 30 °C. The engineered strains were verified by colony PCR. The correct single-colonies were inoculated in 10 mL of YPD medium for one day. 1% of the inoculated volume was transferred and incubated for another day, strewn in YPD plates containing 1 g/L 5-fluoroorotic acid.

### 2.3 Shake flask fermentation and bioreactor fermentation

Single-colonies of *S. cerevisiae* were selected from YNB plates without amino acids, and cultured into a 250 mL shake flask containing 25 mL of yeast extract peptone dextrose (YPD) medium (20 g/L tryptone, 10 g/L yeast extract, and 20 g/L glucose) for 24 h at 30 °C and 220 rpm. The seed solution was inoculated into 25 mL of YPD medium to give an initial optical density (OD) of approximately 0.2. After 120 h of fermentation, the titer was measured using GC-MS.

Fermentation was carried out in a 5 L bioreactor (T&J Bioengineering, Shanghai, China) containing 2.5 L of fermentation medium (30 g/L glucose, yeast extract 10 g/L, tryptone 20 g/L, magnesium sulfate heptahydrate 1.5 g/L, calcium carbonate 10 g/L, 0.01% defoamer). The initial agitation speed was 250 rpm, and the dissolved oxygen (DO) was controlled at 20%–40%. The feed medium (600 g/L glucose, 24 mL/L vitamin solution, and 20 mL/L trace metal solution) was streamed after 12 h in the fermentation process. Then the absolute ethanol was continuously added for 60 h. Trace metal solution and vitamin solution were prepared according to the formula of our previous work [18]. The formulations were also provided in the supplementary data.

### 2.4 Standards and analytical methods

The standard (–)-α-bisabolol was purchased from Sigma-Aldrich (Saint Louis, MO, USA). The mother liquor of (–)-α-bisabolol at a concentration of 10 g/L was obtained by dissolving 1 g of (–)-α-bisabolol standard in 100 mL of ethanol. For analysis, the stock solution of 10 g/L (–)-α-bisabolol in ethanol was diluted into a series of solutions from 5 mg/L to 500 mg/L as the standards.

To extract extracellular (–)-α-bisabolol, 500 μL fermentation culture was mixed with 500 μL methanol. The mixture was vortexed for 1 min and centrifuged. The supernatant was used for analysis after filtration through a 0.22 μm nylon membrane. To extract intracellular (–)-α-bisabolol, cells of 500 μL culture was collected through centrifuging, washed twice with distilled water, and then resuspended into 1 mL ethanol. The cells were then analyzed using a glass bead homogenizer (FastPrep-24 5G). After centrifuging, the supernatant was collected and filtered using a 0.22 μm nylon membrane for the analysis. To quantify (–)-α-bisabolol, the samples were analyzed by using a Shimadzu's Gas Chromatograph Mass Spectrometer QP2010 (Kyoto, Japan). An RTX-5 capillary column (30 m × 0.25 mm × 0.25 μm) was used under the following conditions: initial column temperature at 90 °C for 0.5 min, a heating rate of 20 °C/min to 280 °C for 10 min. The injector temperature was 280 °C. The ion source and interface temperatures were 200 °C and 170 °C respectively. The carrier air helium velocity was 1.6 mL/min, and the injection volume was 1 μL. Total ion chromatographs of the fermentation sample and (–)-α-bisabolol standard in this study are shown in Fig. S1.

## 3 Results

### 3.1 Enhancing (–)-α-bisabolol biosynthesis by weakening *ERG9* expression

**Fig. 2:**
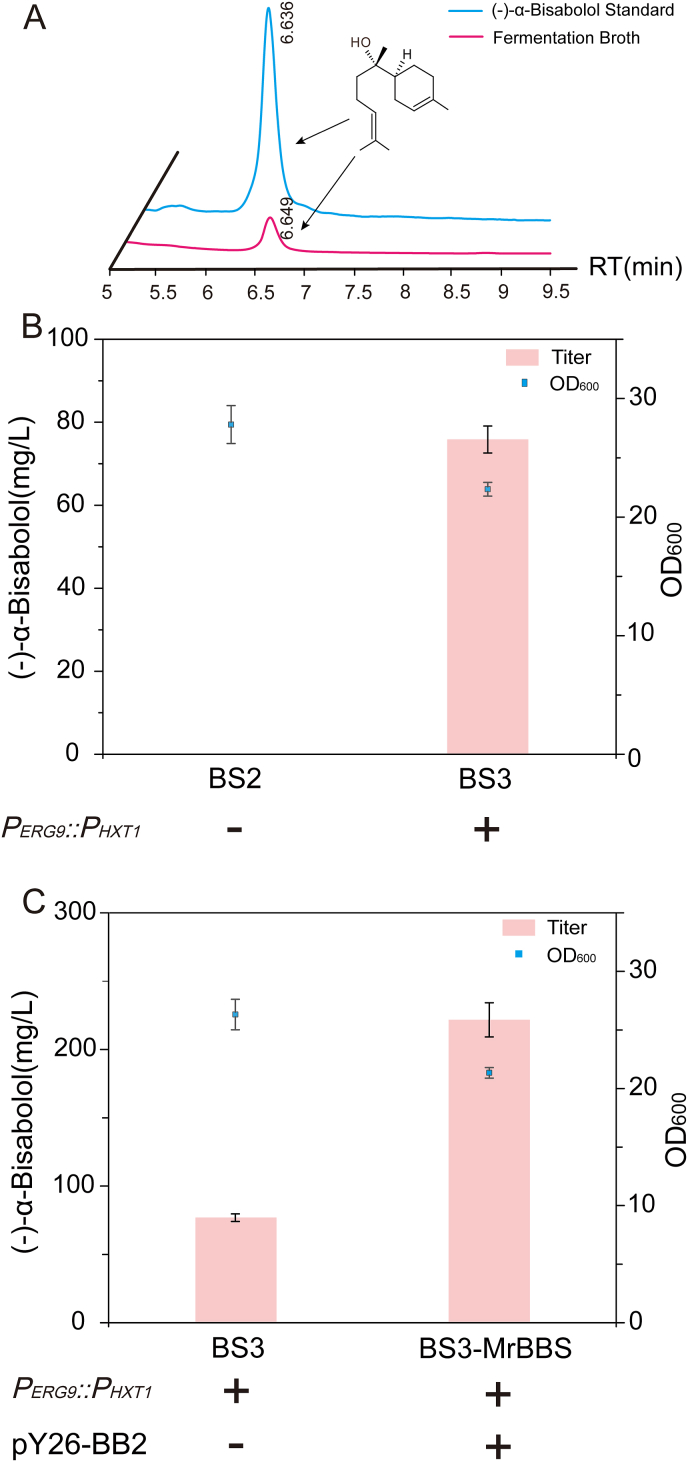
Effect of *ERG9* weakening and the copy number of *MrBBS* on the titer of (–)-α-bisabolol. A. Comparison of peak time of (–)-α-bisabolol standard and peak time of fermentation broth. The gas chromatogram of fermentation broth was marked by the red line, and the (–)-α-bisabolol standard was marked by the blue line. B. Cell growth and production of **(–)-α-bisabolol** after weakening of *ERG9* promoter in strains BS2 and BS3. C. Cell growth and production of (–)-α-bisabolol in strains BS3 and BS3-MrBBS. The values are the average of three biological replicates. Error bars represent standard deviations.

(–)-α-Bisabolol synthase gene from *M. recutita* (*MrBBS*) [7], driven by the *GAL10* promoter, was integrated into the genome of SQ14 to generate strain BS2. However, (–)-α-bisabolol was not detected. *ERG9* is a key gene that determines the metabolic flux of the MVA pathway to ergosterol synthesis (Fig. 1). Therefore, the strain BS3 was obtained by using the low-transcription-level *HXT1* promoter to replace the initial promoter of *ERG9* of strain BS2, and the (–)-α-bisabolol production in shake flask reached 75.83 mg/L (Fig. 2A, B and S1). The OD_600_ of BS3 was decreased by 19.60%, which may be related to the weakening of *ERG9*. Previously, some studies have shown that increasing copy number of *MrBBS* may improve the (–)-α-bisabolol production [10]. We further expressed the (–)-α-bisabolol synthase (*Mr*BBS) using a high-copy 2-μm plasmid pY26-TEF-GPD to generate the strain BS3-MrBBS [19], which could produce 221.96 mg/L of (–)-α-bisabolol (Fig. 2C). In summary, it was found that high copy expression of the heterologous gene and weakening of the *ERG9* gene were the keys to increase the titer of (–)-α-bisabolol.

### 3.2 Fusion expression of *ERG20* and *MrBBS*

**Fig. 3:**
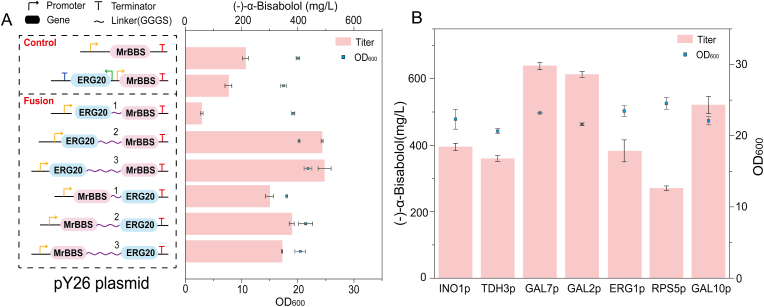
Effects of fusion expression and promoter strength on the yield of the (–)-α-bisabolol. A. Production of (–)-α-bisabolol and cell growth after fusion expression of *ERG20* and *MrBBS*. The numbers ‘1, 2, 3’ represent the copy number of linkers. The yellow arrow represents the *GAL10* promoter and the green arrow represents the *TEF1* promoter. The far left of the gene is the start codon, and the start codon linked to the linker sequence was deleted. The green arrow represents P_*TEF1*_ and the yellow arrow represents P_*GAL10*_. The red ‘T’ represents T_*TAT1*_ and the blue ‘T’ represents T_*ADH1*_. These gene cassettes were constructed in the pY26 plasmid and expressed in BS3. B. Production of (–)-α-bisabolol and cell growth under the control of different strength promoters. The values are the average of three biological replicates. Error bars represent standard deviations.

Both *ERG20* and *MrBBS* are the key genes in the (–)-α-bisabolol synthesis pathway, but their co-expression resulted in a decline in production of (–)-α-bisabolol (Fig. 3A). We speculated that substrate diffusion effects may have reduced reaction efficiency. Fusion expression between enzymes can prevent substrate diffusion effects [20]. The linkers GGGS, (GGGS)_2_, and (GGGS)_3_ were used for fused expression of *ERG20* and *MrBBS* (Fig. 3A). The best combination strain was Erg20 fused with *Mr*BBS by (GGGS)_3_, which produced 496.33 mg/L of **(–)-α-bisabolol** (Fig. 3A).

Promoter strength is directly related to gene transcription level. To further improve the efficiency of Erg20-(GGGS)_3_*-Mr*BBS. The gradient promoters P_*INO1*_, P_*TDH3*_, P_*ERG1*_ and P_*RPS5*_ were selected, and their strengths ranged from strong to weak [19]. Three promoters of the *GAL* series, P_*GAL10*_, P_*GAL7*_ and P_*GAL2*_ were also selected. Among them, P_*GAL7*_ had the best performance, although its intensity was not the highest. The production of (–)-α-bisabolol further increased to 639.34 mg/L (Fig. 3B).

### 3.3 Improving precursor supplementation by modulating the MVA pathway

**Fig. 4:**
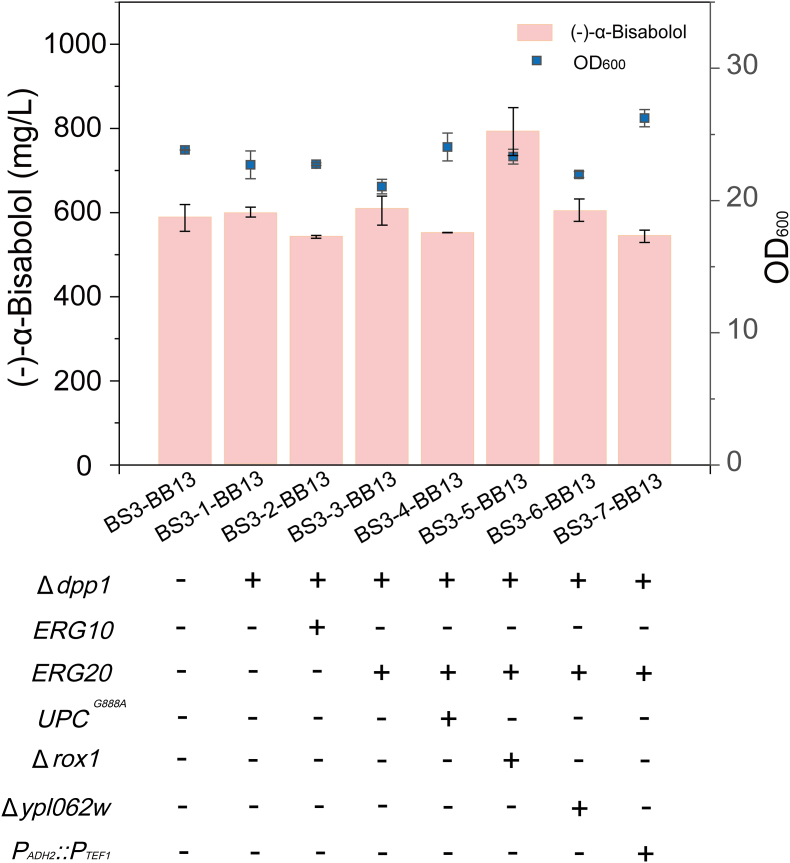
Effects of genes related to FPP generation on the yield of (–)-α-bisabolol Comparison of (–)-α-bisabolol production in engineered recombinant *S. cerevisiae* strains by modulating the MVA pathway (strains all harbor the plasmid pY26-BB13). The values are the average of three biological replicates. Error bars represent standard deviations.

When metabolite flow of the MVA pathway was intensified to **(–)-α-bisabolol**, FPP may become insufficient. Dpp1 is a competitive enzyme that converts FPP to farnesol [21]. Knockout of *DPP1* increased the titer but not significantly. In addition, a key gene *ERG20* was overexpressed at the *DPP1* site, which increased the titer by 3.5% compared with the control (Fig. 4). Overexpression of *ERG10*, another key gene in the MVA pathway, was not ideal for **(–)-α-bisabolol production** (Fig. 4). We speculated that overexpression of a single-gene in the MVA pathway may have caused an accumulation of intermediates. Transcription factor regulation is another solution. Upc2^G888A^ is a global activator of MVA pathway, and its overexpression had been shown to promote the synthesis of trichodermol [22]. Rox1 and Ypl062w were proven to be negative regulators of MVA pathway [23]. Knockout of the *ROX1* showed good results, the titer of **(–)-α-bisabolol** increased to 795.06 mg/L (Fig. 4). In addition, the metabolism of ethanol was enhanced by replacing the wild-type promoter of *ADH2* with P_*TEF1*_. The growth of the resulting strain improved, but (–)-α-bisabolol production was significantly reduced (Fig. 4).

To achieve high copy expression of the key genes at the genomic level, P_*GAL7*_-*ERG20-*(GGGS)_3_*-MrBBS* were integrated at the *Ty3* multi-copy site [24]. After random integration, a genetically stable strain BS5 with a titer of 894.43 mg/L (–)-α-bisabolol was obtained from 12 single-colonies (Fig. S2).

### 3.4 Overexpression of endogenous transporters

**Fig. 5:**
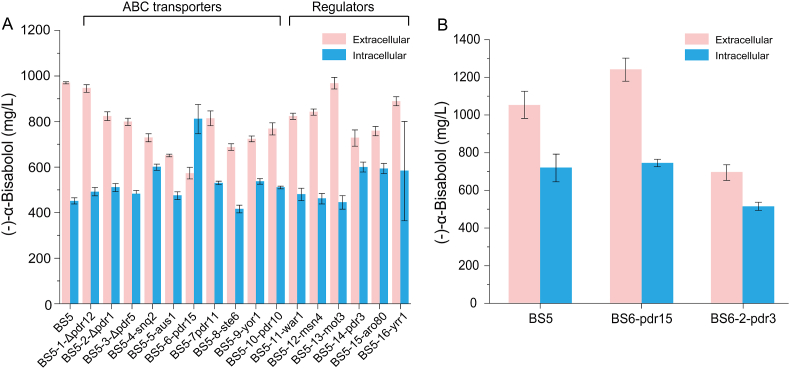
Enhance production of (–)-α-bisabolol by transporter engineering. A. Intracellular and extracellular production of (–)-α-bisabolol after transporters (Δ*PDR12*, Δ*PDR1*, Δ*PDR5*, Δ*SNQ2*, Δ*AUS1*, Δ*PDR15*, Δ*PDR11*, Δ*STE6*, Δ*YOR1*, Δ*PDR10*) and regulators (Δ*WAR1*, Δ*MSN4*, Δ*MOT3*, Δ*PDR3*, Δ*ARO80*, Δ*YRR1*) were knocked out in BS5. B. Intracellular and extracellular production of (–)-α-bisabolol after overexpression of *PDR15* and *PDR3* in BS5. The values are the average of three biological replicates. Error bars represent standard deviations.

Terpenoids are toxic to many host strains, and ATP-binding cassette (ABC) transporters are involved in the transport of the toxic substances [25]. (–)-α-Bisabolol accumulation was detected both inside and outside the cell. This indicates the potential presence of an endogenous transport mechanism for (–)-α-bisabolol to relieve the cytotoxicity in *S. cerevisiae***.** To find an effective transporter, 10 ABC family transporters (*PDR12*, *PDR1*, *PDR5*, *SNQ2*, *AUS1*, *PDR15*, *PDR11*, *STE6*, *YOR1*, and *PDR10*) and 6 transcriptional regulators (*WAR1*, *MSN4*, *MOT3*, *PDR3*, *ARO80*, and *YRR1*) were selected for single-knockout experiments [25,26]. In the strain without *PDR15*, the extracellular (–)-α-bisabolol accumulation was significantly decreased (Fig. 5A), while a large amount of (–)-α-bisabolol was accumulated inside the cell. In addition, compared to other transcription factors, the deletion of *PDR3* also resulted in higher intracellular accumulation. Overexpression of *PDR15* and *PDR3* in BS5 formed BS6 and BS6-2, respectively. Only *PDR15* showed a positive result for (–)-α-bisabolol production (Fig. 5B). The production of extracellular (–)-α-bisabolol increased to 1242.30 mg/L in BS6, while the intracellular yield was close to the control at 745.85 mg/L (Fig. 5B).

### 3.5 Fermentation optimization of high-yielding strains of (–)-α-bisabolol

**Fig. 6:**
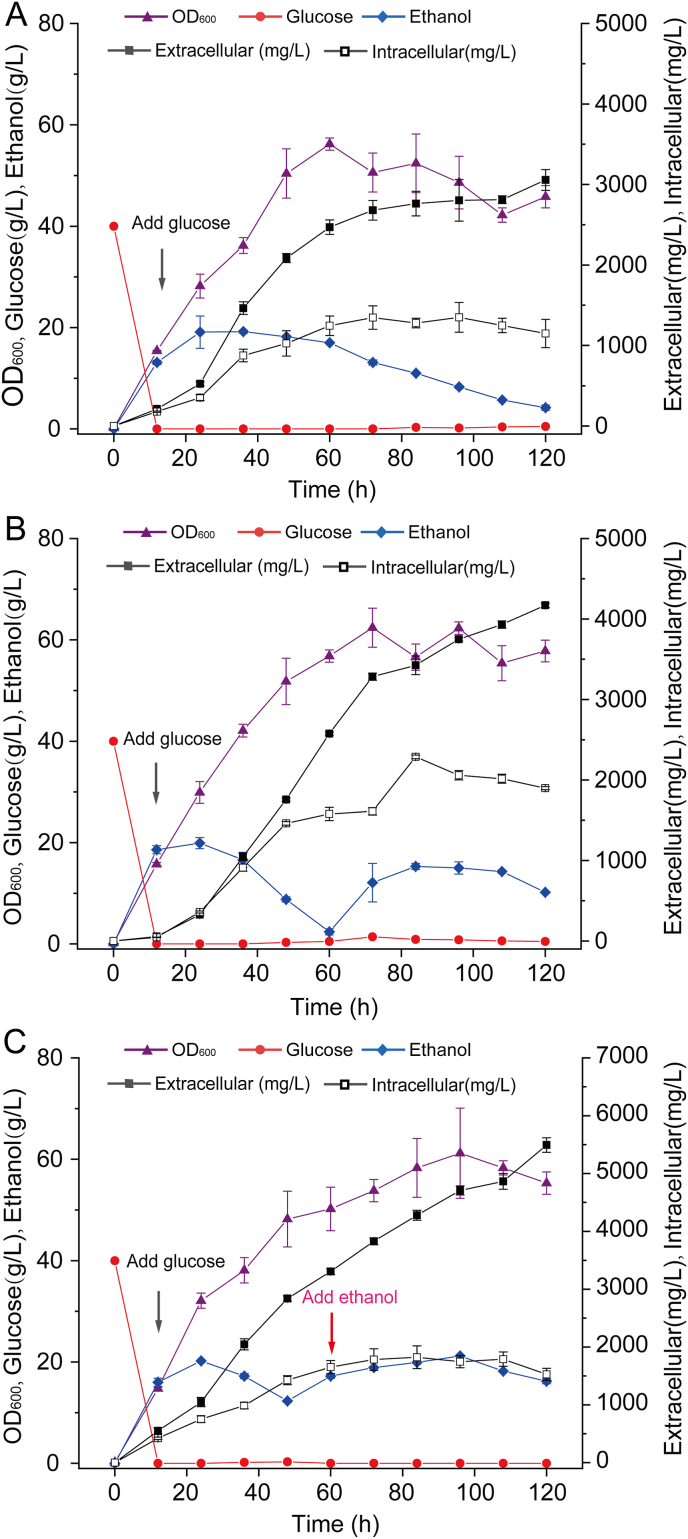
Fed-batch fermentation of (–)-α-bisabolol production in a 5 L bioreactor. Time courses of cell growth, (–)-α-bisabolol production, glucose consumption, and ethanol production are presented. A. Production of (–)-α-bisabolol by batch fermentation in a 5 L bioreactor. The concentration of dissolved oxygen was maintained at 20–30%, and the concentration of glucose was maintained at 0–1 g/L. B. (–)-α-Bisabolol was produced by batch fermentation in a 5 L bioreactor. The dissolved oxygen concentration was maintained at 20–30%, the glucose concentration was maintained at 0–1 g/L, and 10 g/L of calcium carbonate was initially added. C. Production of (–)-α-bisabolol by batch fermentation in a 5 L bioreactor. The dissolved oxygen concentration was maintained at 20–30%, the glucose concentration was maintained at 0–1 g/L, and 10 g/L of calcium carbonate was initially added. Ethanol was added at 60 h, and the concentration of ethanol was kept at 10–20 g/L. The values are the average of three biological replicates. Error bars represent standard deviations.

Stable and appropriate culture conditions are beneficial to the growth of the strain. Moreover, the strategy of feeding affects the economic feasibility of the (–)-α-bisabolol production. Calcium carbonate can maintain the pH relative stability in the fermentation system. Fermentation with glucose and ethanol as the mixed carbon source contributed to the accumulation of terpenoids in *S. cerevisiae* [27]. Preliminary fermentation optimization in the shake flasks revealed that calcium carbonate and ethanol supplements had a positive effect on the synthesis of the product (Fig. S3). To further evaluate the fermentation performance of the strain, a scale-up experiment with a 5 L bioreactor was carried out based on the shake-flask results. First, the influence of calcium carbonate on fermentation was examined (Fig. 6A and B). When calcium carbonate was added, the maximum OD_600_ increased from 56.2 to 62.4, and the yield increased from 4.21 g/L to 6.07 g/L. Compared with the calcium carbonate-free control, the OD_600_ and the yield of (–)-α-bisabolol increased by 11.03% and 44.18%, respectively. These results indicated that the addition of calcium carbonate was beneficial to cell growth and the accumulation of (–)-α-bisabolol. Next, the effects of different carbon sources in the fermentation were further compared. Compared with glucose feeds alone, ethanol significantly enhanced the accumulation of (–)-α-bisabolol, with a yield of 7.02 g/L (Fig. 6B and C). Additionally, the proportion of intracellular production decreased from 31.30% to 21.79%. This result shows that ethanol may promote the transport of (–)-α-bisabolol.

## 4 Discussion and conclusion

(–)-α-Bisabolol has good prospects in health care products and cosmetics. The production of (–)-α-bisabolol by biosynthesis is attractive [28]. In the present study, heterologous production of (–)-α-bisabolol was achieved in *S. cerevisiae*. Weakening of *ERG9* and improving the copy number of *MrBBS* were the keys to (–)-α-bisabolol accumulation. Fusion expression improved the utilization efficiency of key enzymes on substrates. Knockout of the repressive factor Rox1 significantly improved the titer of (–)-α-bisabolol. Overexpression of *PDR15* effectively promoted the efflux of (–)-α-bisabolol. Finally, by optimizing the media composition and feed strategy, we have achieved a titer of 7.02 g/L in a 5 L bioreactor. (–)-α-Bisabolol production in yeast is summarized in Table 1. Our work has value for the study of economically viable (–)-α-bisabolol-producing *S. cerevisiae*.

The formation of efficient metabolic channels by co-localizing enzymes can greatly improve the catalytic activity of key enzymes [29]. Achieving protein assembly through protein ligation is a common strategy [30]. The type and length of the linkers are critical to the effect of protein ligation [31]. Excessively long linkers reduced the stability of fusion proteins, while excessively short linkers affected the correct translation and folding of proteins [32]. In this study, when a single-copy linker was used to connect Erg20 and *Mr*BBS, the yield decreased, while a two-copy linker significantly increased the yield. This indicated that too short a linker may affect the correct expression of Erg20 and *Mr*BBS. Guo et al. explored the effects of different linker lengths on the synthesis of resveratrol from 4CL and *STS* catalytic substrates, which greatly improved its catalytic efficiency [33]. Meanwhile, the direction of the linker also impacts protein expression. A suitable linker helped Erg20 and *Mr*BBS maximize their catalytic activity. Jiang et al. designed the direction of the GES and Erg20 fusion protein with the help of surface electrostatic distribution analysis, which greatly increased the geraniol production [34]. In this work, a fusion of Erg20 and *Mr*BBS improved the metabolic flux toward (–)-α-bisabolol synthesis.

A lack of precursor FPP supply would reduce production efficiency [35]. Main competitive gene *ERG9* which is essential for yeast growth that can only be weakened [36]. Replacing the wild promoter of *ERG9* with weak promoters such as P_*HXT1*_ or weakening the catalytic performance of Erg9 by reducing the half-life of the protein were proven feasible to weaken *ERG9* [15,37]. The strategy of replacing the wild-type promoter of *ERG9* with P_*HXT1*_ also guided our fermentation optimization. In our study, glucose supplementation helped to activate the expression of *ERG9* during the early growth stage of the strain, while ethanol supplementation inhibited the expression of *ERG9* and enhanced the (–)-α-bisabolol production. In addition, the MVA pathway is directly related to FPP production. HmgR catalyzes the conversion of HMG-CoA to mevalonic acid, which has been recognized as the first rate-limiting step of MVA pathway due to the irreversibility of the reaction. Erg20 catalyzes the synthesis of FPP from IPP and DMAPP, and its high expression is beneficial to improve the content of FPP. Meng et al. significantly increased the titer of the precursor valencene by the overexpression of *tHMG1* and the fusion expression of *ERG20* and *CnVS*, laying the foundation for the *de novo* synthesis of nootkatone [38]. The regulation of transcription factors is also an effective strategy. Knockout of *ROX1* increased the transcription level of the whole MVA pathway to enhance the supply of FPP precursors [23], and the (–)-α-bisabolol production was improved significantly. Overexpressing a non-limiting gene alone was not helpful to production, whereas increasing the whole metabolic flux of the MVA pathway was the best choice for (–)-α-bisabolol production.

Extracellular transport engineering can reduce metabolic pressure on cells and significantly increase titer of the products [39]. *S. cerevisiae* contains powerful transport systems that transport a wide variety of toxic substances. ABC transporters have detoxifying properties in prokaryotic and eukaryotic cells and have been shown to export a wide variety of products. Xu et al. improved the efficiency of the extracellular transport of rubusoside by overexpressing the yeast endogenous transporter Pdr11 and transcriptional activator Msn4, thus improving the titer of rubusoside [26]. Jiao et al. simultaneously overexpressed *PDR11* and *YOL075C*, which was favorable for the transport of tocotrienol [40]. Therefore, we tried to explore some highly efficient endogenous ABC transporters. *PDR15* is a paralog of *PDR5*. Both are involved in multi-substrate resistance mechanisms [41]. *PDR15* was shown to be involved in (–)-α-bisabolol transport, with significantly improved the titer after overexpression. The crystal structure and transport mechanism of *PDR5* (the paralog of *PDR15*) have been resolved [42], while most of the other binding transport mechanisms between transporters and substrates have yet to be elucidated.

In summary, a high titer (–)-α-bisabolol-producing strain was constructed by expressing heterologous genes, fusion expression, modulating the MVA pathway, strengthening the transport mechanism and fermentation optimization. The production of (–)-α-bisabolol reached 7.02 g/L in a 5-L bioreactor. After our work, there are still some strategies to enhance (–)-α-bisabolol synthesis. First, *S. cerevisiae* has a strong ethanol biosynthesis pathway, and the supply of acetyl-CoA (precursor of the MVA pathway) may be insufficient. Systematic enhancement of the acetyl-CoA synthesis pathway is an effective approach to improve the production of (–)-α-bisabolol. Second, high expression of the MVA pathway gene consumes large amounts of NADPH, and the supply of cofactor NADPH may also be a limiting factor. Finally, intracellular accumulation of the product remains a problem. Two-phase fermentation may further promote product secretion [40].

## CRediT authorship contribution statement

**Yinkun Jiang:** Methodology, Investigation, Formal analysis, Writing – original draft. **Lu Xia:** Investigation, Formal analysis. **Song Gao:** Formal analysis, Supervision, Designed and supervised the project, Revised the manuscript. **Ning Li:** Supervision, Designed and supervised the project, Revised the manuscript. **Shiqin Yu:** Supervision, Designed and supervised the project, Revised the manuscript. **Jingwen Zhou:** Supervision, Designed and supervised the project, Funding acquisition, Revised the manuscript, All authors discussed the results and made suggestions on the manuscript.

## Declaration of competing interest

The authors declare that they do not have any financial or commercial conflict of interest in connection with the work submitted.
