# Supplementary material for: Engineering Saccharomyces cerevisiae for enhanced (–)-α-bisabolol production
Source: Synth Syst Biotechnol. 2023 Jan 20;8(2):187–95. doi: 10.1016/j.synbio.2023.01.004 (PMC9941373; doi:10.1016/j.synbio.2023.01.004)
Supplement: Multimedia component 1 [file mmc1.docx]

**Supplementary Information**

# Metabolic engineering of *Saccharomyces cerevisiae* for enhanced production of (–)-α-bisabolol

Yinkun Jiang^1,2^, Lu Xia^1,2^, Song Gao^1,2^, Ning Li^1,2^, Shiqin Yu^1,2^, Jingwen Zhou^1,2,3,4^*

^1^ Engineering Research Center of Ministry of Education on Food Synthetic Biotechnology, Jiangnan University, 1800 Lihu Road, Wuxi, Jiangsu 214122, China;

^2^ Science Center for Future Foods, Jiangnan University, 1800 Lihu Road, Wuxi, Jiangsu 214122, China;

^3^ Key Laboratory of Industrial Biotechnology, Ministry of Education and School of Biotechnology, Jiangnan University, 1800 Lihu Road, Wuxi, Jiangsu 214122, China;

^4^ Jiangsu Province Engineering Research Center of Food Synthetic Biotechnology, Jiangnan University, Wuxi 214122, China.

* Correspondence to:

Jingwen Zhou

Science Center for Future Foods, Jiangnan University, 1800 Lihu Rd, Wuxi, Jiangsu 214122, China.

Phone: +86-510-85914371, Fax: +86-510-85914371

E-mail: zhoujw1982@jiangnan.edu.cn

## Formulation of trace solution and vitamin solution

The trace metal solution consisted of 5.75 g/L ZnSO_4_·7H_2_O, 0.32 g/L MnCl_2_·4H_2_O, 0.47 g/L CoCl_2_·6H_2_O, 0.48 g/L Na_2_MoO_4_·2H_2_O, 2.9 g/L CaCl_2_·2H_2_O, 2.8 g/L FeSO_4_·7H_2_O and 80 mL 0.5 M EDTA.

The vitamin solution consisted of 0.05 g/L biotin, 1 g/L calcium pantothenate, 1 g/L nicotinic acid, 25 g/L *myo*-inositol, 1 g/L thiamine HCl, 1 g/L pyridoxal HCl and 0.02 g/L *p*-aminobenzoic acid.

## Supplementary Figures:


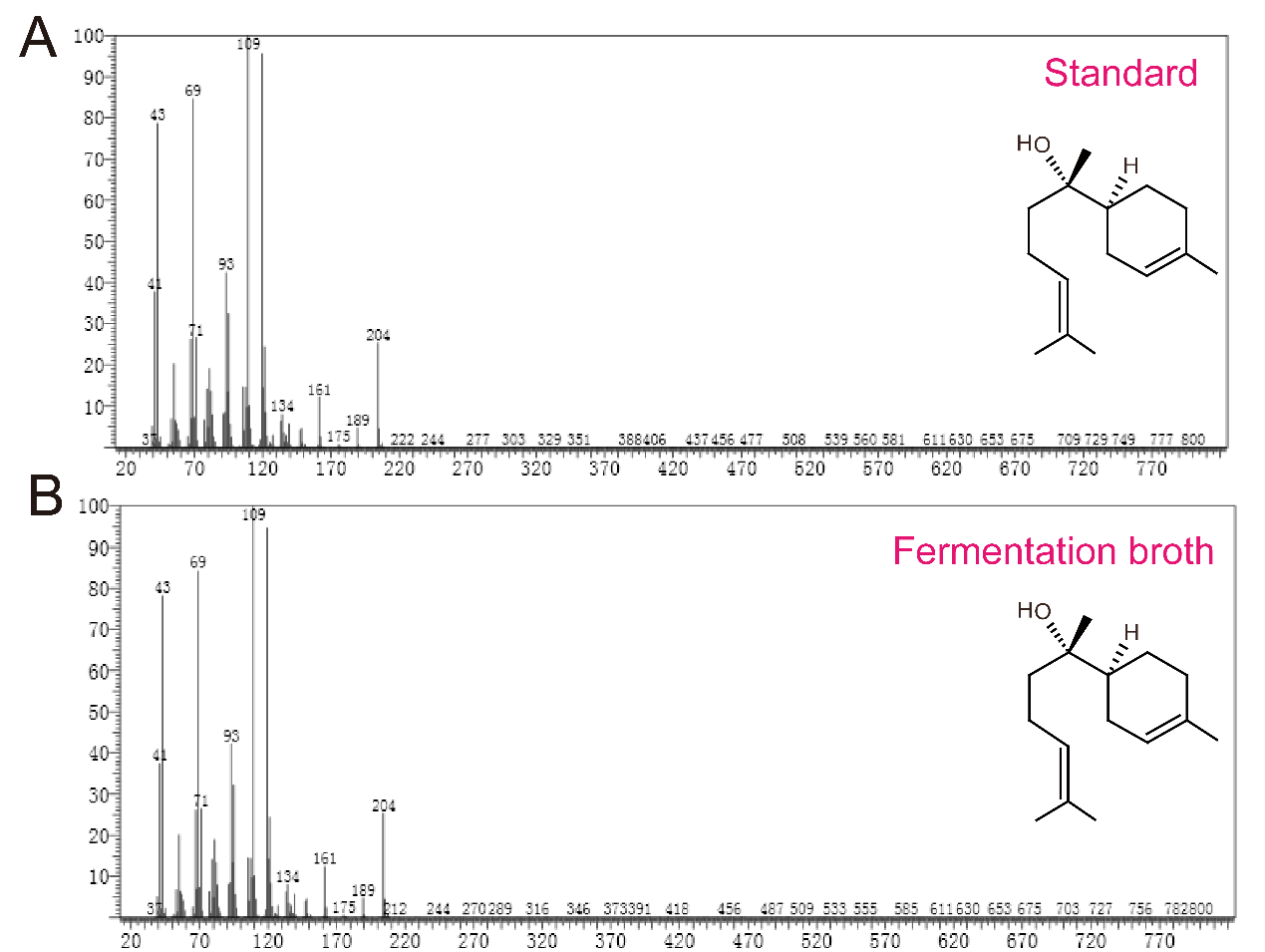


## Supplementary Figure 1. Mass spectra of (–)-α-bisabolol produced by strain BS3 and authentic (–)-α-bisabolol standard. A. (–)-α-Bisabolol standard. B. Fermentation broth.


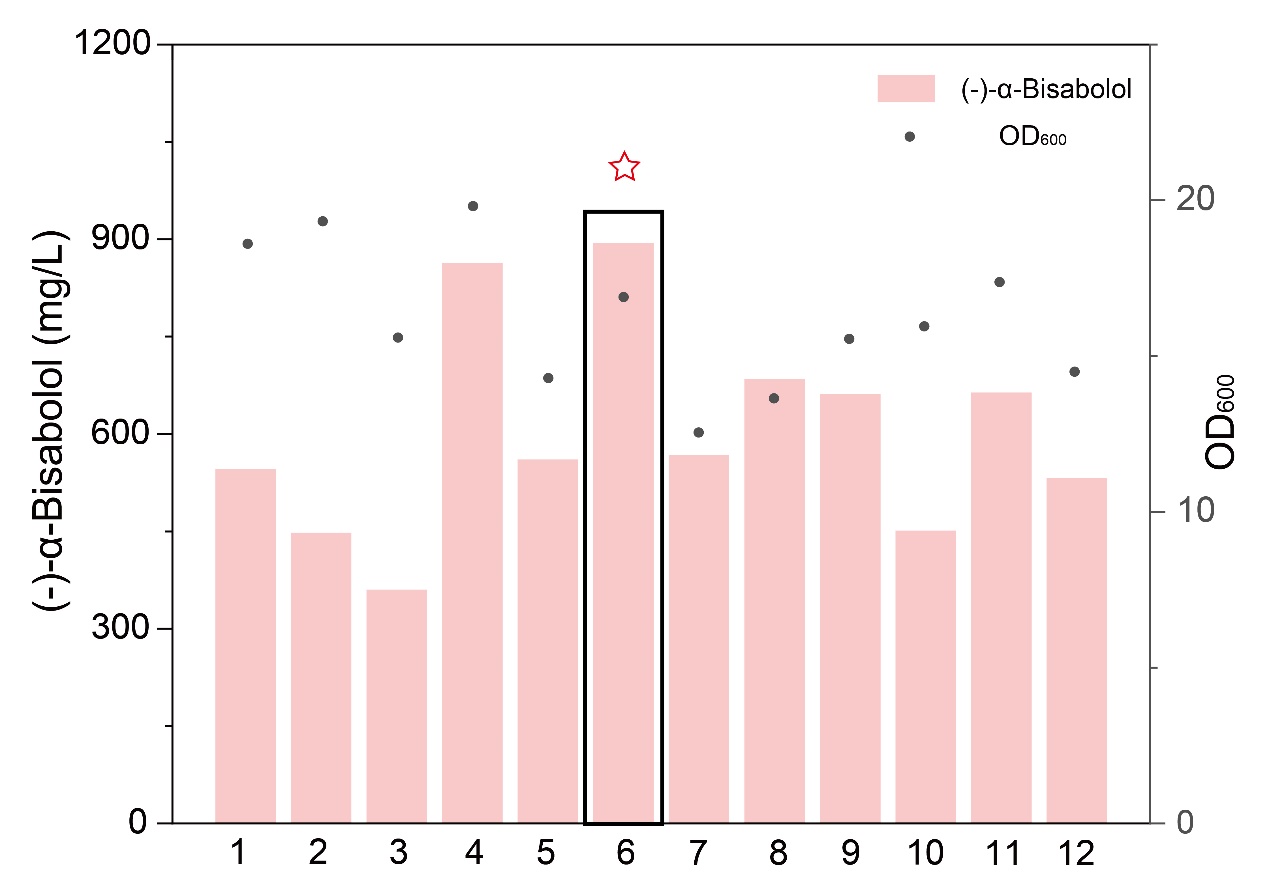


## Supplementary Figure 2. The growth and production of (–)-α-bisabolol by multi-copy site integration. Production of (–)-α-bisabolol and cell growth when the product synthesis module was integrated at the *Ty3* transposon. Twelve individual colonies were randomly selected.


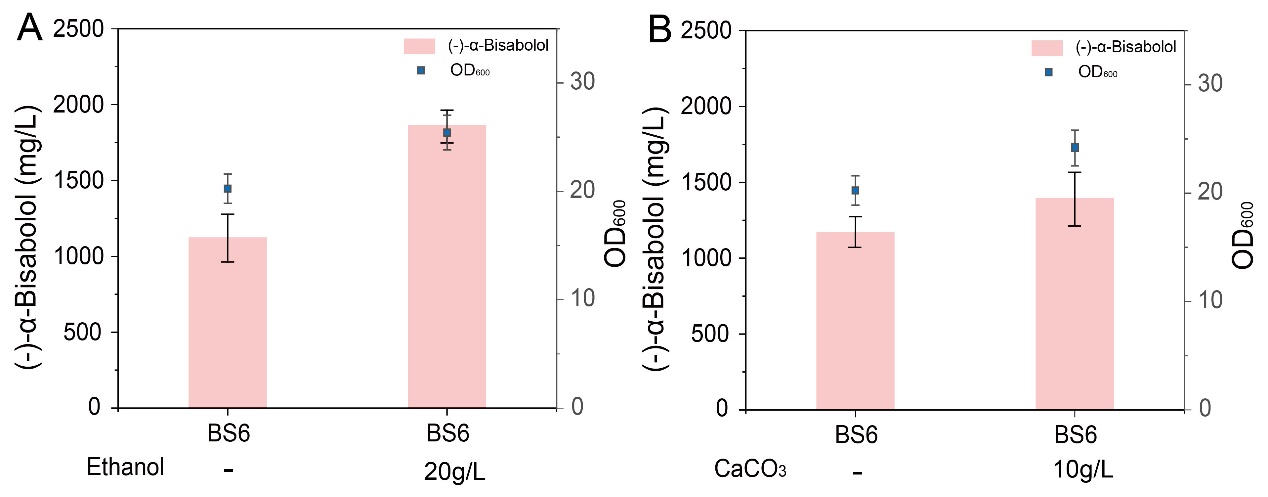


## Supplementary Figure 3. Shake flask optimization. A. 20 g/L of ethanol was added after 24 h of seeding. B. The initial medium was added to 10 g/L of CaCO_3_. The values are the average of three biological replicates. Error bars represent standard deviations.

## Supplementary Tables:

## Table S1. Plasmids used in this study

| **Name** | | **Description** | **Source** |
| --- | --- | --- | --- |
| pRS426 | sgRNA expression plasmid | | [1] |
| pRS426-sg*SPR1* | sgRNA expression plasmid for inserting at *SPR1* | | This study |
| pRS426-sgP*_ERG9_* | sgRNA expression plasmid for inserting at P*_ERG9_* | | This study |
| pRS426-sg*DPP1* | sgRNA expression plasmid for inserting at *DPP1* | | This study |
| pRS426-sg*ROX1* | sgRNA expression plasmid for inserting at *ROX1* | | This study |
| pRS426-sg*YPL062W* | sgRNA expression plasmid for inserting at *YPL062W* | | This study |
| pRS426-sgP*_ADH2_* | sgRNA expression plasmid for inserting at P*_ADH2_* | | This study |
| pRS426-sg*PDR15* | sgRNA expression plasmid for inserting at *PDR15* | | This study |
| pRS426-sg*PDR3* | sgRNA expression plasmid for inserting at *PDR3* | | This study |
| pCT44 | Ty3-P*_GAL7_*-*EGFP*-T*_TAT1_*-*KlURA3deg* | | [2] |
| pY26-TEF-GPD | AmpR, *URA3*, Express vector | | [2] |
| pMD19T-Simple | AmpR, Clone vector | | [3] |
| pMD19T-BB0 | pMD19T-spr1U-P*_GAL10_*-*MrBBS*-T*_TAT1_*-spr1D | | This study |
| pMD19T-BB1 | pMD19T-P*_ERG9_*U-P*_HXT1_*-P*_ERG9_*D | | This study |
| pY26-BB2 | pY26-P*_GAL10_*-*MrBBS*-T*_TAT1_* | | This study |
| pY26-BB3 | pY26-P*_GAL1_*-*ERG20*-P*_GAL10_*-*MrBBS*-T*_TAT1_* | | This study |
| pY26-BB4 | pY26-P*_GAL10_*-*ERG20-*GGGS*-MrBBS*-T*_TAT1_* | | This study |
| pY26-BB5 | pY26-P*_GAL10_*-*MrBBS-*GGGS*-ERG20*-T*_TAT1_* | | This study |
| pY26-BB6 | pY26-P*_GAL10_*-*ERG20-*(GGGS)_2_*-MrBBS*-T*_TAT1_* | | This study |
| pY26-BB7 | pY26-P*_GAL10_*-*ERG20-*(GGGS)_3_*-MrBBS*-T*_TAT1_* | | This study |
| pY26-BB8 | pY26-P*_GAL10_*-*MrBBS*-(GGGS)_2_*-ERG20*-T*_TAT1_* | | This study |
| pY26-BB9 | pY26-P*_GAL10_*-*MrBBS*-(GGGS)_3_-*ERG20*-T*_TAT1_* | | This study |
| pY26-BB10 | pY26-P*_INO1_*-*ERG20*-(GGGS)_3_-*MrBBS-*T*_TAT1_* | | This study |
| pY26-BB11 | pY26-P*_TDH3_*-*ERG20*-(GGGS)_3_-*MrBBS*-T*_TAT1_* | | This study |
| pY26-BB12 | pY26-P*_GAL7_*-*ERG20*-(GGGS)_3_-*MrBBS*-T*_TAT1_* | | This study |
| pY26-BB13 | pY26-P*_GAL2_-ERG20*-(GGGS)_3_-*MrBBS*-T*_TAT1_* | | This study |
| pY26-BB14 | pY26-P*_ERG1_*-*ERG20*-(GGGS)_3_-*MrBBS*-T*_TAT1_* | | This study |
| pY26-BB15 | pY26-P*_RPS5_*-*ERG20*-(GGGS)_3_-*MrBBS*-T*_TAT1_* | | This study |
| pCT44-BB16 | Ty3-P*_GAL7_*-*ERG20*-(GGGS)_3_-*MrBBS*-T*_TAT1_*-*KlURA3deg* | | This study |
| pMD19T-BB17 | pMD19T-dpp1U-dpp1D | | This study |
| pMD19T-BB18 | pMD19T-dpp1U-P*_GAL10_*-*ERG20*-T*_TAT1_*-dpp1D | | This study |
| pMD19T-BB19 | pMD19T-dpp1U-P*_GAL10_*-*ERG10*-T*_TAT1_*-dpp1D | | This study |
| pMD19T-BB20 | pMD19T-*ypl062w*U-*ypl062w*D | | This study |
| pMD19T-BB21 | pMD19T-*rox1*U-*rox1w*D | | This study |
| pMD19T-BB22 | pMD19T-dpp1U-P*_GAL2_*-*UPC2^G888A^*-T*_ADH2_*-P*_GAL10_*-*ERG20*-T*_TAT1_*-dpp1D | | This study |
| pMD19T-BB23 | pMD19T-P*_ADH2_*U-P*_TEF1_*-P*_ADH2_*D | | This study |
| pMD19T-BB24 | pMD19T-pdr12U-*HIS3*-pdr12D | | This study |
| pMD19T-BB25 | pMD19T-pdr1U-*HIS3*-pdr1D | | This study |
| pMD19T-BB26 | pMD19T-pdr5U-*HIS3*-pdr5D | | This study |
| pMD19T-BB27 | pMD19T-snq2U-*HIS3*-snq2D | | This study |
| pMD19T-BB28 | pMD19T-aus1U-*HIS3*-aus1D | | This study |
| pMD19T-BB29 | pMD19T-pdr15U-*HIS3*-pdr15D | | This study |
| pMD19T-BB30 | pMD19T-pdr11U-*HIS3*-pdr11D | | This study |
| pMD19T-BB31 | pMD19T-ste6U-*HIS3*-ste6D | | This study |
| pMD19T-BB32 | pMD19T-yor1U-*HIS3*-yor1D | | This study |
| pMD19T-BB33 | pMD19T-pdr10U-*HIS3*-pdr10D | | This study |
| pMD19T-BB34 | pMD19T-war1U-*HIS3*-war1D | | This study |
| pMD19T-BB35 | pMD19T-msn4U-*HIS3*-msn4D | | This study |
| pMD19T-BB36 | pMD19T-mot3U-*HIS3*-mot3D | | This study |
| pMD19T-BB37 | pMD19T-pdr3U-*HIS3*-pdr3D | | This study |
| pMD19T-BB38 | pMD19T-aro80U-*HIS3*-aro80D | | This study |
| pMD19T-BB39 | pMD19T-yrr1U-*HIS3*-yrr1D | | This study |
| pMD19T-BB40 | pMD19T-pdr15U*-*P*_TEF1_*-*PDR15*-T*_ADH2_*-pdr15D | | This study |
| pMD19T-BB41 | pMD19T-pdr3U*-*P*_TEF1_*-*PDR3*-T*_ADH2_*-pdr3D | | This study |

## Table S2. Gene, promoter, and linker used in this study

| **Name** | **Sequence** | |
| --- | --- | --- |
| *MrBBS* | | ATGTCAACTTTATCAGTTTCTACTCCTTCCTTCTCTTCATCTCCATTGTCTTCGGTTAATAAAAACAGCACGAAACAACACGTTACTCGCAATAGTGTAATCTTCCATGATAGTATCTGGGGGGATCAATTTTTGGAATATAAGGAAAAGTTTAATGTAGCTACTGAGAAACAGCTAATCGAAGAGTTGAAAGAAGAAGTGAGAAACGAATTGATGATCAGAGCTTGTAATGAAGCATCCAGGTATATAAAACTTATCCAATTGATTGATGTAGTTGAAAGGCTTGGTTTGGCCTACCATTTTGAAAAGGAAATCGAGGAATCCTTGCAACATATCTATGTTACATATGGCCATAAATGGACCAACTATAACAACATTGAAAGTCTTTCGCTGTGGTTTAGACTGTTGCGACAAAATGGCTTCAACGTATCATCGGATATATTCGAGAACCATATTGATGAAAAGGGAAATTTCCAGGAAAGCTTATGTAATGACCCTCAAGGGATGCTTGCTTTATACGAAGCAGCATATATGAGGGTTGAAGGTGAGATAATACTAGACAAGGCCTTAGAATTCACCAAATTGCACCTGGGTATCATATCCAATGATCCTTCTTGTGACTCTTCTCTAAGAACAGAAATAAAGCAAGCTCTAAAGCAGCCGCTTCGTAGAAGGTTGCCAAGGCTAGAGGCGGTGCGATACATCGCAATCTATCAACAAAAAGCTTCTCACTCCGAGGTCTTGTTAAAGCTTGCGAAGTTAGACTTTAACGTGTTACAAGAAATGCATAAAGACGAGCTTAGCCAAATTTGCAAATGGTGGAAGGATTTGGACATTAGAAACAAGTTACCCTATGTGCGCGACAGATTGATTGAAGGCTACTTTTGGATCTTGGGAATCTATTTCGAACCCCAACATTCTCGTACAAGAATGTTCTTAATGAAAACATGTATGTGGCTCATTGTTTTAGATGACACGTTTGATAATTATGGTACTTATGAGGAACTTGAAATATTTACACAGGCTGTCGAAAGATGGTCCATAACCTGCTTGGATGAACTACCAGAGTACATGAAGCTAATTTATCATGAGCAGTTCCGTGTTCACCAAGAAATGGAGGAATCACTAGAGAAAGAAGGAAAAGCTTATCAAATACATTATATTAAAGAGATGGCCAAAGAGGGTACAAGGAGCCTTCTGTTAGAAGCCAAATGGTTGAAAGAGGGATACATGCCAACTTTGGATGAGTACCTGTCTAATTCACTAGTTACTTGTGGATATGCACTCATGACAGCCAGATCTTATGTTGCCCGGGATGACGGTATAGTCACGGAGGATGCCTTTAAATGGGTGGCAACTCATCCACCTATTGTCAAAGCTGCATGTAAAATTCTGAGATTAATGGATGATATTGCTACCCACAAGGAGGAACAGGAGAGAGGTCACATTGCTTCAAGTATTGAATGCTACAGAAAGGAAACTGGTGCATCAGAGGAGGAAGCTTGCATGGATTTCTTAAAACAAGTCGAGGATGGTTGGAAGGTTATAAATCAGGAAAGTCTGATGCCTACAGACGTACCATTTCCCCTCCTTATTCCAGCAATTAACCTTGCGCGTGTGAGTGATACCCTATACAAGGATAATGACGGCTACAATCATGCTGATAAAGAAGTCATTGGTTACATCAAATCGTTATTTGTTCACCCGATGATTGTCTAA |
| *PDR15* | | ATGTCATCAGATATCAGAGACGTAGAGGAACGAAATTCGCGGAGCTCGAGCTCAAGCTCGAGCTCGAACTCTGCCGCCCAATCCATTGGACAGCATCCATACCGCGGTTTCGACAGCGAAGCCGCGGAAAGGGTGCATGAGTTGGCTAGAACACTCACATCGCAGAGTTTACTATACACTGCTAACTCAAACAATAGCTCTTCCAGCAACCATAATGCGCACAATGCGGACTCGAGATCCGTATTTTCTACGGACATGGAAGGTGTGAACCCGGTGTTCACTAACCCGGACACCCCGGGATACAATCCCAAATTGGACCCTAACAGTGATCAATTCTCCAGTACGGCGTGGGTACAGAACATGGCAAATATTTGTACTTCGGACCCGGATTTCTATAAACCATACTCACTCGGTTGTGTATGGAAGAATCTAAGCGCCTCCGGAGACTCCGCCGATGTGTCATACCAGTCAACTTTCGCTAACATCGTACCAAAGCTGCTTACGAAAGGGCTCAGATTACTGAAGCCCAGTAAAGAAGAGGACACTTTTCAGATCCTGAAACCCATGGATGGTTGTCTTAATCCCGGTGAACTGTTGGTTGTTCTTGGGAGACCAGGGTCAGGTTGTACTACGCTGCTAAAATCCATATCTTCTAATTCGCACGGGTTCAAGATCGCAAAAGACTCCATTGTCTCTTACAACGGTTTGTCAAGCTCGGATATCAGGAAACATTACCGTGGTGAAGTCGTTTACAATGCGGAATCAGATATTCATTTACCGCATCTTACCGTGTACCAGACGCTTTTCACCGTGGCAAGGATGAAAACGCCGCAAAATCGTATCAAGGGTGTTGATAGAGAAGCGTACGCCAATCACGTGACAGAAGTTGCAATGGCCACATATGGTCTTTCGCATACAAGGGACACCAAGGTCGGAAACGATCTAGTCAGAGGTGTTTCTGGTGGTGAAAGAAAGCGTGTATCCATTGCGGAAGTCGCAATCTGCGGCGCCAGATTTCAATGTTGGGATAATGCCACCAGAGGTTTGGACTCTGCTACTGCTTTAGAATTCATCCGTGCTTTAAAAACCCAGGCTGACATTGGAAAGACCGCGGCTACTGTGGCCATCTATCAATGTTCTCAGGATGCTTATGATCTTTTTGATAAGGTCTGTGTCCTGGATGATGGTTACCAGCTTTATTTTGGGCCCGCTAAGGATGCAAAGAAATATTTCCAAGACATGGGCTACTATTGTCCTCCCAGACAGACCACTGCAGATTTTTTAACTTCAATTACAAGTCCTACTGAAAGAATTATTAGCAAAGAATTTATCGAAAAAGGTACTAGAGTGCCTCAAACGCCAAAGGATATGGCCGAATATTGGCTACAATCAGAAAGCTACAAAAATTTAATCAAGGATATAGACTCTACATTAGAGAAAAACACAGATGAAGCACGCAATATTATCAGGGATGCTCACCACGCTAAACAGGCAAAAAGGGCACCACCTTCCTCTCCATACGTTGTCAACTACGGAATGCAAGTCAAATACTTGTTGATTAGAAATTTCTGGAGAATGAAGCAAAGTGCTAGTGTTACTTTGTGGCAAGTCATCGGTAACTCTGTCATGGCTTTCATCTTGGGTTCTATGTTTTATAAAGTGATGAAGAAAAACGATACTTCCACTTTCTATTTCCGTGGTGCTGCAATGTTTTTTGCTATTTTATTTAATGCATTTTCGTGTCTTTTGGAAATCTTTAGTTTGTATGAAACAAGACCTATAACTGAAAAACACAGAACTTATTCCTTGTATCATCCAAGCGCTGACGCATTTGCGTCTGTTTTGTCAGAAATGCCGCCAAAATTGATCACTGCTGTCTGCTTCAACATCATCTTTTATTTCCTAGTTGATTTCAGGAGAAACGGTGGTGTCTTTTTCTTTTATTTTTTAATTAATGTCATTGCCACATTCACTTTATCCCATTTATTTAGATGCGTGGGCTCCTTGACCAAAACATTACAGGAGGCCATGGTCCCCGCTTCAATGTTATTATTGGCAATTTCTATGTATACAGGGTTTGCCATCCCTAAAACGAAGATTTTAGGTTGGTCCATTTGGATTTGGTATATCAACCCGCTAGCCTACCTGTTTGAATCTTTAATGATCAATGAATTCCATGACCGTAGATTCCCTTGTGCCCAATACATCCCTGCTGGCCCTGCTTATCAGAACATCACAGGTACTCAACGCGTCTGTTCCGCAGTTGGTGCTTATCCAGGTAACGACTATGTGTTGGGTGATGACTTCTTGAAGGAAAGTTACGATTATGAGCACAAACATAAGTGGCGTGGGTTCGGTATTGGTATGGCATATGTTGTTTTCTTTTTCTTTGTTTATCTAATTCTTTGTGAGTATAATGAAGGTGCTAAACAAAAGGGTGAAATGGTTGTGTTCCTAAGATCTAAGATCAAGCAATTGAAAAAGGAAGGTAAATTACAAGAAAAGCATAGGCCTGGAGATATTGAAAATAATGCAGGCAGTTCTCCAGATTCCGCTACAACAGAAAAAAAAATACTAGATGATAGTTCTGAGGGATCGGATAGCTCTTCAGATAATGCCGGATTAGGACTTTCCAAATCTGAAGCAATTTTCCACTGGCGTGATTTATGCTATGATGTTCCTATAAAAGGAGGTCAAAGACGTATCTTAAATAACGTAGATGGTTGGGTAAAACCAGGCACTTTGACTGCCTTAATGGGGGCGTCAGGTGCAGGTAAAACAACTTTACTGGATTGTTTGGCTGAAAGAGTCACCATGGGTGTTATTACTGGTAATATTTTTGTCGATGGTCGTCTCCGTGACGAATCATTCCCTAGATCTATTGGTTATTGTCAACAACAGGATTTACATTTGAAAACGGCTACAGTAAGAGAATCTTTGAGATTTTCAGCTTATTTACGTCAGCCTTCGTCAGTTTCTATTGAAGAAAAAAATAGGTACGTGGAAGAAGTTATCAAAATTTTGGAAATGCAACAATATTCAGATGCTGTTGTTGGTGTTGCAGGTGAAGGTCTAAATGTCGAACAAAGAAAAAGACTTACTATTGGTGTTGAACTAGCGGCAAGGCCTAAACTTTTGGTTTTTTTGGATGAACCAACTTCAGGCCTGGATTCACAAACTGCTTGGGACACTTGCCAACTTATGAGGAAACTAGCTACCCACGGTCAAGCAATTTTGTGTACTATCCATCAACCTTCTGCTATATTAATGCAGCAGTTTGATAGATTATTATTTTTACAGAAAGGGGGCCAAACTGTATATTTCGGCGATTTAGGTGAAGGGTGCAAAACTATGATCGATTATTTTGAAAGTAAAGGAGCTCACAAATGTCCACCTGATGCAAACCCTGCCGAATGGATGTTAGAGGTTGTAGGTGCCGCTCCTGGTTCTCACGCTACGCAAGATTATAATGAAGTCTGGAGAAACTCAGATGAATACAAAGCTGTTCAGGAAGAATTGGATTGGATGGAAAAGAATTTACCAGGCAGGTCAAAAGAACCAACTGCAGAAGAACATAAACCTTTTGCTGCATCTTTATACTACCAATTTAAAATGGTTACCATTCGTTTGTTCCAACAATACTGGAGATCACCAGATTATTTATGGTCGAAATTTATTTTGACTATTTTCAACCAAGTTTTTATTGGGTTCACTTTTTTCAAGGCTGACAGAAGTTTACAGGGACTACAAAACCAAATGTTATCAATATTCATGTATACGGTTATTTTCAATCCTATACTACAACAGTATCTACCATCTTTCGTGCAGCAAAGGGATTTGTATGAGGCAAGAGAACGTCCTTCAAGAACATTTTCGTGGCTTGCGTTTTTCCTCTCTCAAATCATTGTTGAAATCCCATGGAATATTTTAGCGGGTACGATTGCTTATTGTATTTACTATTACGCGGTTGGATTTTATGCGAACGCCTCAGCTGCTGGTCAACTCCATGAGAGAGGTGCTTTGTTTTGGTTATTTTCTATTGCCTTCTACGTCTACATTGGTTCGATGGGTTTGTTAATGATTTCTTTCAATGAAGTTGCTGAAACAGCGGCACATATGGGAACGCTATTGTTCACGATGGCATTATCTTTCTGTGGTGTTATGGCTACCCCTAAGGTTATGCCAAGATTTTGGATCTTTATGTATAGAGTGTCACCCCTAACTTATATGATCGATGCATTATTAGCCCTTGGTGTGGCTAATGTGGACGTTAAGTGTTCAAATTATGAAATGGTAAAATTTACTCCACCATCTGGAACCACCTGCGGTGACTATATGGCATCATATATCAAGTTGGCCGGAACAGGCTACTTGAGTGACCCGTCTGCAACAGATATATGTAGTTTCTGTGCGGTATCCACCACCAATGCCTTTTTGGCCACTTTCAGTTCTCATTATTACAGAAGATGGAGAAATTACGGTATTTTTATCTGCTATATTGCTTTTGATTATATCGCTGCAACATTCTTGTATTGGTTATCCAGGGTACCCAAGAAGAACGGTAAGATTTCCGAAAAACCCAAGAAGTGA |
| *PDR3* | | ATGAAAGTGAAGAAATCAACTAGATCAAAAGTTTCGACAGCATGTGTCAATTGCAGAAAAAGGAAAATCAAATGCACAGGTAAATATCCATGTACCAACTGCATTTCTTACGATTGTACGTGTGTATTCCTAAAAAAACATTTACCGCAGAAGGAGGATAGTTCCCAGTCTTTGCCTACTACAGCTGTTGCTCCACCCTCTTCCCATGCCAATGTAGAGGCTTCGGCAGATGTACAGCATCTGGACACTGCGATTAAGCTAGATAATCAATATTACTTCAAACTGATGAACGACCTGATACAGACTCCAGTCTCTCCGAGTGCGACGCATGCTCCTGATACTTCCAATAATCCTACTAATGATAATAATATTCTCTTTAAAGATGATTCCAAATATCAAAATCAACTGGTTACGTATCAAAATATTCTGACAAATTTGTACGCTCTGCCGCCTTGTGATGACACTCAGCTCTTGATTGATAAAACGAAGTCGCAGTTGAATAACCTGATTAACAGTTGGAATCCCGAAATAAACTACCCCAAGCTTTCCAGTTTCTCTCCTCGCCCACAGAGATCGATAGAAACGTATCTTTTAACCAACAAGTATAGAAATAAAATACACATGACGAGGTTCTCCTTTTGGACAGACCAAATGGTTAAATCACAAAGTCCAGATTCATTTCTAGCCACCACTCCACTAGTAGATGAAGTATTTGGTCTTTTCTCTCCAATACAGGCTTTTTCACTAAGAGGTATAGGATATTTAATTAAAAAAAATATCGAAAACACGGGTTCATCGATGTTAATAGATACAAAGGAAACTATTTATCTAATATTAAGATTGTTCGATTTGTGTTATGAACATTTGATCCAAGGTTGCATCTCTATTTCTAATCCATTAGAGAACTATCTTCAAAAAATAAAGCAAACTCCTACTACGACGGCATCTGCTAGTTTGCCTACTTCCCCAGCACCTTTATCTAACGATTTAGTCATTTCTGTTATTCATCAACTACCTCAGCCATTTATACAATCGATTACCGGGTTTACGACTACTCAATTGATAGAAAATTTACATGATTCATTTTTGATGTTTCGAATAGTTACTCAAATGTATGCTCAACATAGGAAGCGCTTTGCGGAATTTTTAAACCAGGCTTTCTCCTTGCCCCATCAAGAAAAGAGTGTTTTATTCTCATCATTCTGCTCATCAGAATATCTTCTATCTACTCTTTGTTACGCATACTACAATGTTACCCTATATCACATGTTAGACATAAACACTTTAGATTACCTAGAGATTTTAGTGTCATTGCTAGAAATCCAAAATGAAATTGATGAGCGTTTTGGATTTGAAAAAATGCTAGAAGTTGCGGTTACATGCTCCACTAAGATGGGATTGTCTCGTTGGGAGTATTATGTTGGAATAGACGAAAATACTGCCGAACGGAGAAGAAAAATATGGTGGAAAATATACAGTCTGGAAAAGCGTTTTTTAACTGATCTTGGTGATTTATCCTTAATAAATGAACATCAAATGAATTGTCTCTTGCCGAAGGATTTCAGGGACATGGGATTCATTAACCATAAAGAATTTTTAACGAAAATTGGTACGTCCTCTTTATCACCGTCATCGCCCAAGCTAAAAAATTTGTCATTGTCCAGGCTTATTGAATATGGTGAGTTAGCGATAGCCCAAATTGTTGGAGATTTTTTTTCAGAGACTCTCTATAATGAGAAATTCACATCTTTAGAAGTATCCGTTAAACCCACAATTATCAGACAAAAGTTATTGGAGAAAGTTTTTGAGGACATTGAATCTTTTAGGTTAAAATTGGCCAAAATAAAGCTTCACACCTCAAGAGTTTTTCAAGTAGCTCACTGCAAATATCCAGAATATCCAAAAAACGATTTAATTGAAGCAGCTAAATTTGTAAGTTACCATAAAAATACATGGTTCTCCATCTTGGGTGCTGTTAACAATCTTATTGCTAGGCTATCTGAAGATCCAGAGGTGATAACTGAGCAAAGCATGAAATATGCGAATGAAATGTTTCAAGAATGGAGGGAAATTAATCAATTCTTAATACAGGTTGATACTGATTTTATTGTTTGGGCATGTTTGGACTTTTATGAACTGATATTTTTCGTGATGGCTTCAAAATTTTATGTGGAAGACCCGCACATCACTTTAGAGGATGTTATCAACACTTTGAAAGTTTTTAAGAGAATAACTAACATTATTTCTTTTTTTAATAATAATTTGGACGAGAAGGATTATGATTGTCAAACTTTCAGGGAGTTTTCGAGAAGTTCGAGTTTGGTTGCCATATCCATAAGAATCATATTTTTAAAATACTGCTATGCCGAACAAATTGATAGAGCCGAATTCATCGAACGTTTGAAAGAAGTTGAACCGGGTCTAAGTGACCTTTTGCGTGAGTTTTTTGATACCCGCTCTTTTATTTACAGGTACATGTTGAAATCCGTTGAAAAATCAGGCTTTCATTTAATAATTAGAAAAATGTTAGAAAGCGACTATAAATTTTTGTATAGAGACAAATTGGCCACTGGTAATATTCCAGATCAAGGAAATTCAAGCCAAATTTCTCAGTTGTATGACAGTACTGCTCCTTCATACAACAATGCTTCTACCTCAGCAGCAAATTCACCGTTGAAGTTATCGTCTTTGTTGAACTCTGGAGAGGAATCGTACACTCAAGACGCATCAGAAAATGTTCCATGTAATCTGCGGCATCAAGATCGATCGTTACAACAGACAAAAAGACAACATTCTGCGCCTAGCCAAATAAGCGCTAATGAGAATAATATATACAACTTGGGTACTTTAGAGGAGTTTGTTAGCAGTGGTGACCTGACTGATTTATATCATACTCTGTGGAATGACAATACTTCATATCCCTTCTTATGA |
| *DPP1* | | ATGAACAGAGTTTCGTTTATTAAAACGCCTTTCAACATAGGGGCGAAATGGAGATTAGAAGATGTCTTTTTGCTCATTATCATGATACTTCTTAACTACCCAGTGTATTACCAACAACCGTTCGAACGTCAGTTTTACATTAACGATCTCACTATATCGCATCCTTATGCGACAACTGAACGTGTAAATAACAACATGTTGTTTGTTTATAGTTTTGTCGTGCCATCTTTAACCATATTGATAATTGGTTCCATTTTGGCCGATAGAAGACATTTGATTTTTATTTTGTACACATCTCTCCTTGGTTTATCACTCGCTTGGTTCAGTACGAGTTTCTTTACAAACTTCATCAAGAATTGGATTGGAAGACTAAGACCAGATTTTCTAGATCGTTGCCAACCTGTTGAAGGCTTGCCATTGGACACTTTATTTACTGCAAAAGATGTGTGTACGACTAAGAATCACGAACGTCTGTTGGATGGGTTTAGGACAACTCCGTCAGGTCATTCAAGTGAAAGCTTTGCAGGACTGGGTTATTTGTACTTCTGGCTATGTGGGCAACTTTTGACTGAATCACCGTTGATGCCTTTATGGAGAAAAATGGTGGCCTTTCTACCACTGTTAGGAGCTGCACTAATTGCTCTATCCAGAACTCAAGATTACAGACATCATTTCGTCGATGTAATTTTAGGGTCTATGTTGGGTTATATAATGGCACACTTTTTCTACAGAAGAATCTTCCCACCCATTGATGATCCTCTTCCGTTCAAACCATTGATGGACGATTCAGATGTCACCCTGGAGGAAGCAGTCACCCATCAGAGGATCCCGGATGAGGAATTACATCCTTTGTCCGATGAAGGTATGTAA |
| *ROX1* | | ATGAATCCTAAATCCTCTACACCTAAGATTCCAAGACCCAAGAACGCATTTATTCTGTTCAGACAGCACTACCACAGGATCTTAATAGACGAATGGACCGCTCAAGGTGTGGAAATACCCCATAATTCAAACATTTCTAAAATTATTGGTACGAAGTGGAAGGGCTTACAACCGGAAGATAAGGCACACTGGGAAAATCTAGCGGAGAAGGAGAAACTAGAACATAAAAGGAAGTATCCTGAATACAAATACAAGCCGGTAAGAAAGTCTAAGAAGAAGCAACTACTTTTGAAGGAAATCGAGCAACAGCAGCAGCAGCAACAGAAAGAACAGCAGCAGCAGAAACAGTCACAACCGCAATTACAACAGCCCTTTAACAACAATATAGTTCTTATGAAAAGAGCACATTCTCTTTCACCATCTTCCTCGGTGTCAAGCTCGAACAGCTATCAGTTCCAATTGAACAATGATCTTAAGAGGTTGCCTATTCCTTCTGTTAATACTTCTAACTATATGGTCTCCAGATCTTTAAGTGGACTACCTTTGACGCATGATAAGACGGCAAGAGACCTACCACAGCTGTCATCTCAACTAAATTCTATTCCATATTACTCAGCTCCACACGACCCTTCAACGAGACATCATTACCTCAACGTCGCTCAAGCTCAACCAAGGGCTAACTCGACCCCTCAATTGCCCTTTATTTCATCCATTATCAACAACAGCAGTCAAACACCGGTAACTACAACTACCACATCCACAACAACTGCGACATCTTCTCCTGGGAAATTCTCCTCTTCTCCGAACTCCTCTGTACTGGAGAACAACAGATTAAACAGTATCAACAATTCAAATCAATATTTACCTCCCCCTCTATTACCTTCTCTGCAAGATTTTCAACTGGATCAGTACCAGCAGCTAAAGCAGATGGGACCAACTTATATTGTCAAACCACTGTCTCACACCAGGAACAATCTATTGTCCACAACTACCCCTACGCATCATCACATTCCTCATATACCAAACCAAAACATTCCTCTACATCAAATTATAAACTCAAACAACACTGAGGTCACCGCTAAAACTAGCCTAGTTTCTCCGAAATGA |
| *YPL062W* | | ATGATAGAATTGGATTATGTAAAAGGTGAAGATACCATTGTAGAAGCAACCAGCACGTCGCCGTGGCTGATGAGGTCTCCTCTTGCCCGGGCCGCAGAAAAGAGGGGCAGTGGCCTGTTTTTCGACATAAATGAGGGGCATGGCCAGCACCGAGACGTCATTGTTGCATATGGCGTATCCAAGCCGAAACGGCGCTCGCCTCATCCCCACGGGAATAAGGCAGCCGACAAAAGAAAAACGACCGAAAAGGAACCAGAAAGAAAAAAGAGGGTGGGCGCGCCGCGGACGTGTAAAAAGATATGCATCCAGCTTCTATATCGCTTTAACTTTACCGTTTTGGGCATCGGGAACGTATGTAACATTGATCTCCTCTTGGGAACGGTGAGTGCAACGAATGCGATATAG |
| *UPC^G888A^* | | ATGAGCGAAGTCGGTATACAGAATCACAAGAAAGCGGTGACAAAACCCAGAAGAAGAGAAAAAGTCATCGAGCTAATTGAAGTGGACGGCAAAAAGGTGAGTACGACTTCAACCGGTAAACGTAAATTCCATAACAAATCAAAGAATGGGTGCGATAACTGTAAAAGAAGAAGAGTTAAGTGTGATGAAGGGAAGCCAGCCTGTAGGAAGTGCACAAATATGAAGTTGGAATGTCAGTATACACCAATCCATTTAAGGAAAGGTAGAGGAGCAACAGTAGTGAAGTATGTCACGAGAAAGGCAGACGGTAGCGTGGAGTCTGATTCATCGGTAGATTTACCTCCTACGATCAAGAAGGAGCAGACACCGTTCAATGATATCCAATCAGCGGTAAAAGCTTCAGGCTCATCCAATGATTCCTTTCCATCAAGCGCCTCTACAACTAAGAGTGAGAGCGAGGAAAAGTCATCGGCCCCTATAGAGGACAAAAACAATATGACTCCTCTAAGTATGGGCCTCCAGGGTACCATCAATAAGAAAGATATGATGAATAACTTTTTCTCTCAAAATGGCACTATTGGTTTTGGTTCTCCTGAAAGATTGAATTCAGGTATCGATGGCTTACTATTACCGCCATTGCCTTCTGGAAATATGGGTGCGTTCCAACTTCAGCAACAGCAGCAAGTGCAGCAGCAATCTCAACCACAGACCCAAGCGCAGCAAGCAAGTGGAACTCCAAACGAGAGATATGGTTCATTCGATCTTGCGGGTAGTCCTGCATTGCAATCCACGGGAATGAGCTTATCAAATAGTCTAAGCGGGATGTTACTATGTAACAGGATTCCTTCCGGCCAAAACTACACTCAACAACAATTACAATATCAATTACACCAGCAGCTGCAATTGCAACAGCATCAGCAAGTTCAGCTGCAGCAGTATCAACAATTACGTCAGGAACAACACCAACAAGTTCAGCAACAACAACAGGAACAACTCCAGCAATACCAACAACATTTTTTGCAACAGCAGCAACAAGTACTGCTTCAGCAAGAGCAACAACCTAACGATGAGGAAGGTGGCGTTCAGGAAAAAAACAGCAAAAAGGTAAAGGAAGGGCCTTTACAATCACAAACAAGCGAAACTACTTTAAACAGCGATGCTGCTACATTACAAGCTGATGCATTATCTCAGTTAAGTAAGATGGGGCTAAGCCTAAAGTCGTTAAGTACCTTTCCAACAGCTGGTATTGGTGGTGTTTCCTATGACTTTCAGGAACTGTTAGGTATTAAGTTTCCAATAAATAACGGCAATTCAAGAGCTACTAAGGCCAGCAACGCAGAGGAAGCTTTGGCCAATATGCAAGAGCATCATGAACGTGCAGCTGCTTCTGTAAAGGAGAATGATGGTCAGCTCTCTGATACGAAGAGTCCAGCGCCATCGAATAACGCCCAAGGGGGAAGTGCTAGTATTATGGAACCTCAGGCGGCTGATGCGGTTTCGACAATGGCGCCTATATCAATGATTGAAAGAAACATGAACAGAAACAGCAACATTTCTCCATCAACGCCCTCTGCAGTGTTGAATGATAGGCAAGAGATGCAAGATTCTATAAGTTCTCTAGGAAATCTGACAAAAGCAGCCTTGGAGAACAACGAACCAACGATAAGTTTACAAACATCACAGACAGAGAATGAAGACGATGCATCGCGGCAAGACATGACCTCAAAAATTAATAACGAAGCTGACCGAAGTTCTGTTTCTGCTGGTACCAGTAACATCGCTAAGCTTTTAGATCTTTCTACCAAAGGCAATCTGAACCTGATAGACATGAAACTGTTTCATCATTATTGCACAAAGGTCTGGCCTACGATTACAGCGGCCAAAGTTTCTGGGCCTGAAATATGGAGGGACTACATACCGGAGTTAGCATTTGACTATCCATTTTTAATGCACGCTTTGTTGGCATTCAGTGCCACCCATCTTTCGAGGACTGAAACTGGACTGGAGCAATACGTTTCATCTCACCGCCTAGACGCTCTGAGATTATTAAGAGAAGCTGTTTTAGAAATATCTGAGAATAACACCGATGCGCTAGTTGCCAGCGCCCTGATACTAATCATGGACTCGTTAGCAAATGCTAGTGGTAACGGCACTGTAGGAAACCAAAGTTTGAATAGCATGTCACCAAGCGCTTGGATCTTTCATGTCAAAGGTGCTGCAACAATTTTAACCGCTGTGTGGCCTTTGAGTGAAAGATCTAAATTTCATAACATTATATCTGTTGATCTTAGCGATTTAGGCGATGTCATTAACCCTGATGTTGGAACAATTACTGAATTGGTATGTTTTGATGAAAGTATTGCCGATTTGTATCCTGTCGGCTTAGATTCGCCATATTTGATAACACTAGCTTATTTAGATAAATTGCACCGTGAAAAAAACCAGGGTGATTTTATTCTGCGGGTATTTACATTTCCAGCATTGCTAGACAAGACATTCCTGGCATTACTGATGACAGGTGATTTAGGTGCAATGAGAATTATGAGATCATATTATAAACTACTTCGAGGATTTGCCACAGAGGTCAAGGATAAAGTCTGGTTTCTCGAAGGAGTCACGCAGGTGCTGCCTCAAGATGTTGACGAATACAGTGGAGGTGGTGCTATGCATATGATGCTAGATTTCCTCGGTGGCGGATTACCATCGATGACAACAACAAATTTCTCTGATTTTTCGTTATGA |
| P*_HXT1_* | | TAAGGAGTATTGGTTTCGAAAGTTTTTCCGAAGCGGCATGGCAGGGACTACTTGCGCATGCGCTCGGATTATCTTCATTTTTGCTTGCAAAAACGTAGAATCATGGTAAATTACATGAAGAATTCTCTTTTTTTTTTTTTTTTTTTTTTTTTTACCTCTAAAGAGTGTTGACCAACTGAAAAAACCCTTCTTCAAGAGAGTTAAACTAAGACTAACCATCATAACTTCCAAGGAATTAATCGATATCTTGCACTCCTGATTTTTCTTCAAAGAGACAGCGCAAAGGATTATGACACTGTTGCATTGAGTCAAAAGTTTTTCCGAAGTGACCCAGTGCTCTTTTTTTTTTTCCGTGAAGGACTGACAAATATGCGCACAAGATCCAATACGTAATGGAAATTCGGAAAAACTAGGAAGAAATGCTGCAGGGCATTGCCGTGCCGATCTTTTGTCTTTCAGATATATGAGAAAAAGAATATTCATCAAGTGCTGATAGAAGAATACCACTCATATGACGTGGGCAGAAGACAGCAAACGTAAACATGAGCTGCTGCGACATTTGATGGCTTTTATCCGACAAGCCAGGAAACTCCACCATTATCTAATGTAGCAAAATATTTCTTAACACCCGAAGTTGCGTGTCCCCCTCACGTTTTTAATCATTTGAATTAGTATATTGAAATTATATATAAAGGCAACAATGTCCCCATAATCAATTCCATCTGGGGTCTCATGTTCTTTCCCCACCTTAAAATCTATAAAGATATCATAATCGTCAACTAGTTGATATACGTAAAATC |
| P*_ERG9_* | | CCCATCTTCAACAACAATACCGACTTACCATCCTATTTGCTTTGCCCTTTTTCTTTTCCACTGCACTTTGCATCGGAAGGCGTTATCGGTTTTGGGTTTAGTGCCTAAACGAGCAGCGAGAACACGACCACGGGCTATATAAATGGAAAGTTAGGACAGGGGCAAAGAATAAGAGCACAGAAGAAGAGAAAAGACGAAGAGCAGAAGCGGAAAACGTATACACGTCACATATCACACACACACA |
| P*_GAL10_* | | TATAGTTTTTTCTCCTTGACGTTAAAGTATAGAGGTATATTAACAATTTTTTGTTGATACTTTTATGACATTTGAATAAGAAGTAATACAAACTGAAAATGTTGAAAGTATTAGTTAAAGTGGTTATGCAGCTTTTCCATTTATATATCTGTTAATAGATCAAAAATCATCGCTTCGCTGATTAATTACCCCAGAAATAAGGCTAAAAAACTAATCGCATTATCATCCTATGGTTGTTAATTTGATTCGTTAATTTGAAGGTTTGTGGGGCCAGGTTACTGCCAATTTTTCCTCTTCATAACCATAAAAGCTAGTATTGTAGAATCTTTATTGTTCGGAGCAGTGCGGCGCGAGGCACATCTGCGTTTCAGGAACGCGACCGGTGAAGACGAGGACGCACGGAGGAGAGTCTTCCGTCGGAGGGCTGTCGCCCGCTCGGCGGCTTCTAATCCGTACTTCAATATAGCAATGAGCAGTTAAGCGTATTACTGAAAGTTCCAAAGAGAAGGTTTTTTTAGGCTAAGATAATGGGGCTCTTTACATTTCCACAACATATAAGTAAGATTAGATATGGATATGTATATGGTGGTAATGCCATGTAATATGATTATTAAACTTCTTTGCGTCCATCCAAAAAAAAAGTAAGAATTTTTGAAAATTCAATATAA |
| P*_INO1_* | | GAAGACGATGAGGCCGGTGCCGATGTGCCCTTGATGGACAACAAACAACAGCTCTCTTCCGGCCGTACTTAGTGATCGGAACGAGCTCTTTATCACCGTAGTTCTAAATAACACATAGAGTAAATTATTGCCTTTTTCTTCGTTCCTTTTGTTCTTCACGTCCTTTTTATGAAATACGTGCCGGTGTTCCGGGGTTGGATGCGGAATCGAAAGTGTTGAATGTGAAATATGCGGAGGCCAAGTATGCGCTTCGGCGGCTAAATGCGGCATGTGAAAAGTATTGTCTATTTTATCTTCATCCTTCTTTCCCAGAATATTGAACTTATTTAATTCACATGGAGCAGAGAAAGCGCACCTCTGCGTTGGCGGCAATGTTAATTTGAGACGTATATAAATTGGAGCTTTCGTCACCTTTTTTTGGCTTGTTCTGTTGTCGGGTTCCTAATGTTAGTTTTATCCTTGATTTATTCTGTTTCATTCCCTTTTTTTTCCAGTGAAAAAGAAGTAACA |
| P*_TDH3_* | | ATAAAAAACACGCTTTTTCAGTTCGAGTTTATCATTATCAATACTGCCATTTCAAAGAATACGTAAATAATTAATAGTAGTGATTTTCCTAACTTTATTTAGTCAAAAAATTAGCCTTTTAATTCTGCTGTAACCCGTACATGCCCAAAATAGGGGGCGGGTTACACAGAATATATAACATCGTAGGTGTCTGGGTGAACAGTTTATTCCTGGCATCCACTAAATATAATGGAGCCCGCTTTTTAAGCTGGCATCCAGAAAAAAAAAGAATCCCAGCACCAAAATATTGTTTTCTTCACCAACCATCAGTTCATAGGTCCATTCTCTTAGCGCAACTACAGAGAACAGGGGCACAAACAGGCAAAAAACGGGCACAACCTCAATGGAGTGATGCAACCTGCCTGGAGTAAATGATGACACAAGGCAATTGACCCACGCATGTATCTATCTCATTTTCTTACACCTTCTATTACCTTCTGCTCTCTCTGATTTGGAAAAAGCTGAAAAAAAAGGTTGAAACCAGTTCCCTGAAATTATTCCCCTACTTGACTAATAAGTATATAAAGACGGTAGGTATTGATTGTAATTCTGTAAATCTATTTCTTAAACTTCTTAAATTCTACTTTTATAGTTAGTCTTTTTTTTAGTTTTAAAACACCAAGAACTTAGTTTCGAATAAACACACATAAACAAACAAA |
| P*_GAL7_* | | TTTGCCAGCTTACTATCCTTCTTGAAAATATGCACTCTATATCTTTTAGTTCTTAATTGCAACACATAGATTTGCTGTATAACGAATTTTATGCTATTTTTTAAATTTGGAGTTCAGTGATAAAAGTGTCACAGCGAATTTCCTCACATGTAGGGACCGAATTGTTTACAAGTTCTCTGTACCACCATGGAGACATCAAAAATTGAAAATCTATGGAAAGATATGGACGGTAGCAACAAGAATATAGCACGAGCCGCGGAGTTCATTTCGTTACTTTTGATATCACTCACAACTATTGCGAAGCGCTTCAGTGAAAAAATCATAAGGAAAAGTTGTAAATATTATTGGTAGTATTCGTTTGGTAAAGTAGAGGGGGTAATTTTTCCCCTTTATTTTGTTCATACATTCTTAAATTGCTTTGCCTCTCCTTTTGGAAAGCTATACTTCGGAGCACTGTTGAGCGAAGGCTCATTAGATATATTTTCTGTCATTTTCCTTAACCCAAAAATAAGGGAAAGGGTCCAAAAAGCGCTCGGACAACTGTTGACCGTGATCCGAAGGACTGGCTATACAGTGTTCACAAAATAGCCAAGCTGAAAATAATGTGTAGCTATGTTCAGTTAGTTTGGCTAGCAAAGATATAAAAGCAGGTCGGAAATATTTATGGGCATTATTATGCAGAGCATCAACATGATAAAAAAAAACAGTTGAATATTCCCTCAAAA |
| P*_GAL2_* | | TCTCCTACAATACCAGTTTCGCTGCAGAAGGCACATCTATTACATTTACTGAGCATAACGGGCTGTACTAATCCAAGGAGGTTTACGGACCAGAGGAACTTTCCAGATTCAGATCACAGCAATATAGGACTGGAAAACATCAGGTAGCCGCACTCAACTTGTAACTGGCAACTACTTTGCATTAAACTCCAATTAAATGCGGTAGAATCTTTTCAGAAAAGGTATTCAACGTCAATTCGGAAAGCTTCCTTCCGGAATGGCTTAAGTAGGTTGCAATTTCTTTTTCTATTAGTAGCTAAAAATGGGTCACGTGATCTATATTCGAAAGGGGCGGTTGCCTCAGGAAGGCACCGGCGGTCTTTCGTCCGTGCGGAGATATCTGCGCCGTTCAGGGGTCCATGTGCCTTGGACGATATTAAGGCAGAAGGCAGTATCGGGGCGGATCACTCCGAACCGAGATTAGTTAAGCCCTTCCCATCTCAAGATGGGGAGCAAATGGCATTATACTCCTGCTAGAAAGTTAACTGTGCACATATTCTTAAATTATACAATGTTCTGGAGAGCTATTGTTTAAAAAACAAACATTTCGCAGGCTAAAATGTGGAGATAGGATTAGTTTTGTAGACATATATAAACAATCAGTAATTGGATTGAAAATTTGGTGTTGTGAATTGCTCTTCATTATGCACCTTATTCAATTATCATCAAGAATAGCAATAGTTAAGTAAACACAAGATTAACATAATAAAAAAAATAATTCTTTCATA |
| P*_ERG1_* | | TGCGATACTGCCGTAGCGGGCCTTCGTATAGCTCGGCCGAGCTCGTACAAAAGGCAAGCAGTGTATCGGACAGAGCTGATATAACACAATACGCTCGTAGTCGATGCATGCCGTGGCTGCTCTCGGTCGGGTATAAGTCTTAGACAATAGTCTTACCTCGCATGTATAATAAATCTTTTGTATTTAATCTATTATATGTTTCTATGCTTTTTTTTCCTATTGTTGTTTGCTTTTCCTTTTCCTTATTTCTTTCTAGCTTCTAATTTTCTTTCTTTTTTTTTTTTTTTTCATTGAAAATTATATATATATATATATATCAGAACAATTGTCCAGTATTGAACAATACAGGTTATTTCGAACAATTGAAAAAAAAAAATCACAGAAAAACATATCGAGAAAAGGGTC |
| P*_RPS5_* | | ATTTCACGAAAACACCTTGTAAATACTACTTGTGAATATGCAAATAGGAAATAGATATATGCATACAAATAAGAAACAATGAACAGCCTTGAGTTCTCAAATTTGCATATCATTTGTTACGTAATTCCTTATCAAGACCTTTTATACAACAACACCCATATACCCATAATTATTCTTAACCCATACATATTTGTCTCAAGTTTATTTTTCAGATGAGAATGGCCCTGCATCAGTTTTTTTCCAGAAACTAATTCATGGTAACAAACATTTCGCGCCCTGCTACGGGGAAGGAGAATTCTTTTTCTCCCGCCCAGAACGTAAAGCCTTGTTTCTTTCTACCGTACTGGGCCCGTCTAATTCTGCCCCTTTGCTGGGAAATCTCCTTTGCAAGCTCCCCTACCTTCGCCGCAGGCTTAGTGGAGGTCTCACTGAACCCTTCCACCTTGGGCCAACTTCTACGCTCACGTTAGTAGGTAATGGATTATCCACAATGTGCCCATTAGTTCTAAAATATTTTGTACTTTTTATCAATACTTAAAATTTCATTTTGAATTAAAATTACTTTTTATTTTAATTACACTCCTTTCATAATACCAAGAAAAGAGACTAGAAATAACCGACCATTCCAAAG |
| P*_ADH2_* | | TGTTTGTTTGAAGAGACTAATCAAAGAATCGTTTTCTCAAAAAATTTAATATCTTAACTGATAGTTTGATCAAAGGGGCAAAACGTAGGGGCAAACAAACGGAAAAATCGTTTCTCAAATTTTCTGATGCCAAGAACTCTAACCAGTCTTATCTAAAAATTGCCTTATGATCCGTCTCTCCGGTTACAGCCTGTGTAACTGATTAATCCTGCCTTTCTAATCACCATTCTAATGTTTTAATTAAGGGATTTTGTCTTCATTAACGGCTTTCGCTCATAAAAATGTTATGACGTTTTGCCCGCAGGCGGGAAACCATCCACTTCACGAGACTGATCTCCTCTGCCGGAACACCGGGCATCTCCAACTTATAAGTTGGAGAAATAAGAGAATTTCAGATTGAGAGAATGAAAAAAAAAAAAAAAAAAGGCAGAGGAGAGCATAAAAATGGGGTTCACTTTTTGGTAAAGCTATAGCATGCCTATCACATATAAATAGAGTGCCAGTAGCGACTTTTTTCACACTCGAAATACTCTTACTACTGCTCTCTTGTTGTTTTTATCACTTCTTGTTTCTTCTTGGTAAATAGAATATCAAGCTACAAAAAGCATACAATCAACTATCAACTATTAACTATATCGTAATACACA |
| P*_TEF1_* | | GAGCTCATAGCTTCAAAATGTTTCTACTCCTTTTTTACTCTTCCAGATTTTCTCGGACTCCGCGCATCGCCGTACCACTTCAAAACACCCAAGCACAGCATACTAAATTTCCCCTCTTTCTTCCTCTAGGGTGTCGTTAATTACCCGTACTAAAGGTTTGGAAAAGAAAAAAGAGACCGCCTCGTTTCTTTTTCTTCGTCGAAAAAGGCAATAAAAATTTTTATCACGTTTCTTTTTCTTGAAAATTTTTTTTTTGATTTTTTTCTCTTTCGATGACCTCCCATTGATATTTAAGTTAATAAACGGTCTTCAATTTCTCAAGTTTCAGTTTCATTTTTCTTGTTCTATTACAACTTTTTTTACTTCTTGCTCATTAGAAAGAAAGCATAGCAATCTAATCTAAGTTTTCTAGAACTAGT |
| GGGS_1_ | | GGTGGCGGATCT |
| GGGS_2_ | | GGTGGCGGATCTGGTGGCGGATCT |
| GGGS_3_ | | GGTGGCGGATCTGGTGGCGGATCTGGTGGCGGATCT |

# [References](javascript:;)

[1] C. C. Sun, G. J. Li, H. B. Li, Y. B. Lyu, S. Q. Yu, J. W. Zhou. Enhancing Flavan-3-ol Biosynthesis in *Saccharomyces cerevisiae*. J Agric Food Chem, 69 (2021) 12763-12772, 10.1021/acs.jafc.1c04489.

[2] S. Gao, H. R. Zhou, J. W. Zhou, J. Chen. Promoter-Library-Based Pathway Optimization for Efficient (2S)-Naringenin Production from p-Coumaric Acid in *Saccharomyces cerevisiae*. J Agric Food Chem, 68 (2020) 6884-6891, 10.1021/acs.jafc.0c01130.

[3] L. Xia, Y. Lv, S. Liu, S. Yu, W. Zeng, J. Zhou. Enhancing Squalene Production in *Saccharomyces cerevisiae* by Metabolic Engineering and Random Mutagenesis. Front Chem Eng, 3 (2022), 10.3389/fceng.2021.790261.
